# The role of category ambiguity in normal and impaired lexical processing: can you *paint* without the *paint*?

**DOI:** 10.3389/fnhum.2023.1028378

**Published:** 2023-05-04

**Authors:** Sladjana Lukic, Alexandra Krauska, Masaya Yoshida, Cynthia K. Thompson

**Affiliations:** ^1^Department of Communication Sciences and Disorders, Ruth S. Ammon College of Education and Health Sciences, Adelphi University, Garden City, NY, United States; ^2^Department of Linguistics, University of Maryland, College Park, College Park, MD, United States; ^3^Department of Linguistics, Northwestern University, Evanston, IL, United States; ^4^Aphasia and Neurolinguistics Laboratory, Department of Communication Sciences and Disorders, Northwestern University, Evanston, IL, United States

**Keywords:** lexicon, zero-derivation, conversion, grammatical category, morphosyntax, agrammatic aphasia

## Abstract

**Introduction:**

Many words are categorially ambiguous and can be used as a verb (*to paint*) or as a noun (*the paint*) due to the presence of unpronounced morphology or “zero morphology”. On this account, the verb “paint” is derived from the noun “paint” through the addition of a silent category-changing morpheme. Past studies have uncovered the syntactic and semantic properties of these categorially ambiguous words, but no research has been conducted on how people process them during normal or impaired lexical processing. Are these two different uses of “paint” processed in the same way? Does this morphosyntactic structure have an effect on online sentence processing?

**Methods:**

This study presents two experiments that investigate the effect of morphosyntactic complexity in categorially ambiguous words presented in isolation (experiment 1) and in a sentential context (experiment 2). The first experiment tested the ability to process categorially unambiguous and ambiguous nouns and verbs in 30 healthy older adults and 12 individuals with aphasia, using a forced choice phrasal-completion task, in which individuals choose whether *the* or *to* is most compatible with target words.

**Results:**

Healthy controls and individuals with fluent aphasia all showed: (1) a bias toward the base category in selection rates for *the* and *to*, where *the* was selected more frequently for words identified to be base nouns, and *to* was selected more frequently for base verbs, and (2) longer reaction times for ambiguous (over unambiguous) words. However, individuals with non-fluent agrammatic aphasia showed a base-category effect only for nouns, with chance performance for verbs. The second experiment, using an eye-tracking while reading paradigm with 56 young healthy adults, showed a reading time slowdown for derived forms (*to paint*) compared to their base-category counterparts (*the paint*) in sentence contexts.

**Discussion:**

These findings suggest that categorially ambiguous words likely share a common root, and are related by zero-derivation, and that impaired access to the base-category (i.e., verbs like *to visit*) precludes associated morphological processes and therefore the retrieval of the derived-category (i.e., nouns like *the visit*) in non-fluent agrammatic aphasia. This study provides insights into the theory of zero morphology, and the principles that need to be accounted for in models of the lexicon.

## 1. Introduction

There are a number of words in English which are ambiguous. Words like *bat* have multiple semantic representations, and could refer to the animal, the sports equipment, or a swat. Words like *paper* have multiple senses, either as the thin sheets that can be written on, or as a newspaper. There is another group of words that are ambiguous in terms of their syntactic category, such as *visit* or *paint*. It is equally plausible to use *visit* as a verb (*I plan to visit my friend*) and as a noun (*I planned a visit with my friend*). The representation of these categorially ambiguous words has been subject to much debate in the literature. According to some theories, these words would be treated as two separate lexical entries that are related only by overlapping phonology and meaning (Jackendoff, 1976; Aronoff, 1993), or in frameworks such as Distributed Morphology (Halle et al., 1993; Halle and Marantz, 1994), as a single root which attaches to a noun categorizer or verb categorizer. Alternatively, this kind of category ambiguity can be represented as a process of “zero derivation,” where there is a single lexical entry that is derived to another category without overt morphology (Taft and Forster, 1975; Nunberg, 1979), or in Distributed Morphology as a single root which attaches first to one categorizer and then derived to the other category by another categorizer (Harley, 2009).

Many studies on lexical retrieval and sentence processing have focused on syntactic category and meaning ambiguity, but very few have considered the role that morphosyntactic complexity might play in the retrieval and processing of categorially ambiguous words. Importantly, research on syntactic category impairments in language disorders such as post-stroke aphasia has primarily used words that are unambiguously categorized as nouns or verbs, assessed by overt tasks such as a picture naming task. These limitations impede our understanding of the linguistic mechanisms that underlie such deficits. In this study, we investigate the status of categorially ambiguous words by examining whether the morphosyntactic complexity of these words impacts retrieval in healthy individuals and individuals with aphasia, both with fluent and non-fluent agrammatic aphasia, and also observe the reading behavior of healthy individuals when the categorially ambiguous words are presented in a sentence context. This study provides key evidence to suggest that these words are related by zero derivation, rather than constituting separate entries in the lexicon or a shared “root” which receives only one nominal categorizer or verbal categorizer.

The theory of zero derivation suggests that these categorially ambiguous words are related by a morphological process that converts the category of a word without a visible change in form (Lipka, 1986; Don, 2005, among others)^1^. Consequently, they are either grammatically converted from a noun (*the base category*) to become a verb (*the derived category*) (e.g., [_N_ *paint*] –> [_V_ *paint*]), or from a verb to become a noun (e.g., [_V_ *visit*] –> [_N_ *visit*]). The derived form of a verb like *paint* can be represented as [_V_ [_N_ *paint*] -ø], where a zero-morpheme (ø) signals the category change. This suggestion is in line with Single Entry (Nunberg, 1979) and Fully Decompositional (Taft and Forster, 1975) accounts, which suggest that only base categories are stored in the lexicon, and grammatical operations derive one category from the other on-line. The theory of zero derivation would also be compatible with morphosyntactic theories where the lexicon includes fully abstract roots that would undergo derivation through the addition of “categorizers,” according to some accounts in Distributed Morphology (Halle et al., 1993; Halle and Marantz, 1994; Harley, 2009).

The directionality of the derivation can be determined based on both semantic and syntactic analyses of the ambiguous words. Clark and Clark (1979) argue that nouns can surface as verbs via semantic dependencies as shown by paraphrase tests, arguing that the meaning of the derived verb must be explained with the help of the base noun. Accordingly, the verb form *to paint* necessarily implies the use of *a paint*, while *to visit* does not involve the use of *a visit* (similar to word pairs that undergo overt derivations, such as *hospitalize ∼ hospital*, in contrast, to *depart* ∼ *department*). Similarly, Marchand (1969) identifies ways to establish which nouns are derived from verbs: the meaning of the noun *visit* necessarily implies a visiting event, while the noun *paint* does not involve a painting event. These semantic relationships indicate that *paint* is a *base noun* and *visit* is a *base verb*. Moreover, researchers have argued that verb-derived nouns like *a visit* are simple result nominal rather than argument structure nominals (as shown by their inability to take aspectual modifiers, *the visit of the daughter for 3 h vs. *they are visiting the daughter for 3 h*; see Grimshaw, 1990; Borer, 2013), and that noun-derived verbs like *to paint* are mostly transitive, unlike deadjectival verbs like *to clear* that can alternate between transitive and unaccusative argument structures (as shown by their inability to have a thematic patient as its subject, *the wall painted versus *the screen cleared*; Hale and Keyser, 1997; see Method Section “2.2. Materials and procedure”). These paraphrase tests suggest that category information must be relevant to the derivation and lexical representation of categorially ambiguous words, either in the semantic domain or in the syntactic domain (or both, if the semantic complexity is also reflected in the morphosyntactic complexity).

An alternate approach, the Dual Entry hypothesis, suggests that the two words are members of two different lexical entries, linked only by phonology and semantic similarity [in line with Full Listing accounts by Jackendoff (1976, 2002); Amorphous Morphology theories by Anderson (1992) and Aronoff (1993)]. These two separate entries would compete with each other for activation using distributed lexical representations, informed by the syntactic and conceptual context, as discussed by models such as the Interactive Activation Model (McClelland and Rumelhart, 1981) and other distributed models of speech perception (Joordens and Besner, 1994; Gaskell and Marslen-Wilson, 1997). However, this theory has no way to account for the observed syntactic properties of these words or the semantic entailment between the base and derived forms discussed above.

These two theories make different predictions for how categorially ambiguous words are processed, as a consequence of their representation in the lexicon. In the theory where the ambiguous words are stored as separate lexical entries linked only by their identical phonological forms and similar meanings, both [_V_*visit*] and [_N_ *visit*] should be treated similarly in processing, all other processing factors (such as frequency, form typicality, and category-based properties such as event structure, argument structure, etc.) being held equal. Thus, to access either form would incur a similar process of lexical retrieval. In contrast, in the theory where [_N_ *visit*] is derived from [_V_ *visit*] through zero-derivation, the derived category ([_N_ [_V_ *visit*) -ø]) should involve an additional step of morphosyntactic structure building, incurring a greater processing cost relative to the base category. Observing the processing behavior for these categorially ambiguous words in healthy individuals and in individuals with fluent and non-fluent agrammatic aphasia whose patterns of noun/verb production diverge allows a direct comparison of these theories of the lexicon.

Past psycholinguistic studies have examined processing of syntactic category and general ambiguity, assuming that lexical representations are specified for syntactic category. Evidence for this comes from healthy individuals who have shown differential processing of words in different syntactic categories, such as unambiguously used nouns and verbs, observing that more time is required to process verbs than nouns (Kostić and Katz, 1987; Spenney and Haynes, 1989; Frost et al., 1997; Sereno and Jongman, 1997; Deutsch et al., 1998; Sereno, 1999; Monaghan et al., 2003; Kauschke and von Frankenberg, 2008; Cordier et al., 2013). Similarly, past studies on ambiguity processing have shown a processing cost associated with ambiguous words, specifically, an overall advantage for polysemous words (like “paper”) and a disadvantage for homonyms (like “bat”) (see Eddington and Tokowicz, 2015 for review; Lukic et al., 2019). Moreover, across different tasks, greater competition and processing costs have been observed for ambiguous words when the two meanings correspond to the same syntactic category (noun-noun homonyms) compared to when they correspond to different syntactic categories (noun-verb homonyms) (Seidenberg et al., 1982; Mirman et al., 2010). This suggests that the relationship between different meanings of an ambiguous word can influence how easily those meanings can be accessed.

Other studies have investigated the processing of ambiguous words in sentence contexts. Duffy et al. (1988) observed that when the sentence context prior to an ambiguous word does not provide any cues for its interpretation, readers exhibit longer reading times for the ambiguous words. This suggests that when an ambiguous word is encountered in a sentence, all of the available meanings of the word compete for selection; the longer reading time corresponds to the time it takes for one meaning to “win.” When the prior context provides disambiguating cues, the ambiguous words exhibit the same reading times as unambiguous control words, suggesting that multiple interpretations are not activated. The ambiguous words tested in the Duffy et al. (1988) study were ambiguous in meaning, with both interpretations corresponding to the same syntactic category. To further investigate the role that the syntactic analysis has in determining the correct interpretation of ambiguous words, Folk and Morris (2003) looked at the reading times of ambiguous words within the same syntactic category (*calf*) and ambiguous words across categories (*duck*) when they appeared in disambiguating sentence contexts. This study observed longer reading times for ambiguous words within the same syntactic category, but not for ambiguous words in different syntactic categories. The authors argued that the syntactic context helped to eliminate the effect of ambiguity for words like *duck*, where the category information would point to only one interpretation.

Neuropsychological studies also have reported differential processing of words belonging to different categories in neurologically impaired individuals with aphasia, such as difficulties in accessing either nouns or verbs (see Vigliocco et al., 2011 for a review). Specifically, within the aphasia literature, several studies showed that individuals with non-fluent agrammatic aphasia evince deficits in verb production (Baxter and Warrington, 1985; McCarthy and Warrington, 1985; Thompson et al., 2012), whereas those with fluent anomic aphasia show deficits in noun production (Miceli et al., 1984; Miceli and Caramazza, 1988; Zingeser and Berndt, 1990; Silveri and Di Betta, 1997; Rapp and Caramazza, 1998; also see Lukic et al., 2021 on evidence from neurodegenerative disorders). These effects have been attributed to differences in lexical-semantics and morphosyntactic representations between nouns and verbs. Very few studies have examined syntactic category ambiguity in aphasia. In an early study, Caramazza and Hillis (1991) tested the spoken and written retrieval of noun-verb homonyms within a sentence context (e.g., *There’s a crack in the mirror*; *Don’t crack the nuts in here*). They found a deficit in verb retrieval in two participants (phonological in one and orthographic in the other) for noun–verb homonyms when the context required a verb, but not when it required a noun. Similarly, in another study, individuals with non-fluent agrammatic aphasia showed a specific impairment in selecting the contextually appropriate reading of noun-verb ambiguities (Hagoort, 1993). Additionally, Goldberg and Goldfarb (2005) investigated different noun/verb retrieval patterns in individuals with fluent and non-fluent aphasia using noun-verb homonyms in three syntactic contexts (e.g., *Squash and beans* vs. *Squash the bug* / *They burn the toast* vs. *They toast the winner* / *They saw her crash at the corner*, where *crash* is categorially ambiguous). By comparing noun-verb homonym pairs with related meanings (e.g., *to crash/the crash*) to noun-verb homonym pairs with unrelated meanings (e.g., *to squash/the squash*), they found different noun/verb selection patterns but no effect of meaning relatedness. Lastly, studies using eye-tracking to investigate the processing of lexical ambiguity in individuals with aphasia suggested that they have intact lexical access processes, but have specific impairments in lexical integration and/or reanalysis processes (Laurinavichyute et al., 2014; see Sharma et al., 2021 for a review). Specifically, individuals with non-fluent agrammatic aphasia have performed at chance when processing sentences that involved reanalysis (e.g., object relatives “*This is the man that the boy catches”* and sentences with lexical ambiguity “*The PEN is always packed with wooly sheep”*) (Friedmann and Gvion, 2003).

However, these previous studies on categorially ambiguous words in both normal and impaired lexical processing have not considered the morphosyntactic and/or semantic complexity associated with the two forms of the ambiguous word. Therefore, the different processing profiles for the ambiguous words used in these studies could be explained by their semantic or conceptual representations, and/or morphosyntactic complexity. It is unknown whether ambiguous verbs, for example, will behave the same way as unambiguous verbs in on-line processing. Furthermore, how morphosyntactic complexity affects lexical retrieval within the context of aphasia is yet to be explored; it is not yet known if individuals with aphasia exhibit sensitivity to the base category, as expected in healthy individuals, or if they exhibit aphasia-subtype specific deficits (i.e., equal retrieval impairments for both noun and verb forms of an ambiguous verb). Study of categorically ambiguous words will account for the role of both morphosyntactic structures (noun/verb derivatives) and lexical-semantic properties (object/action association) in word retrieval deficits. Thus, we focus on charting in detail the lexical retrieval difficulty in specifically non-fluent agrammatic aphasia by (1) replicating disproportional word-class deficits observed for unambiguous items using a covert production task, and (2) indicating that impaired access to the base form (i.e., verbs, as in *visit*) should prevent retrieval of the derived form (i.e., noun) in categorially ambiguous words, demonstrating the existence of unique morphosyntactic representations for these pairs.

The present study tests for base-category bias effects as well as a processing cost for derived categories during the processing of categorially ambiguous words. These items were balanced on a number of features, including semantic similarity, item, and phrasal frequencies, form typicality, and length. Specifically, we examined whether healthy individuals and individuals with fluent and non-fluent agrammatic aphasia would show a base-category bias (e.g., a noun-based bias for *a paint*; a verb-based bias for *to visit*) during single-word lexical access (experiment 1) and whether healthy individuals would show a processing cost for the derived category (like *to paint*) compared to their base-category counterparts (like *a paint*) during on-line sentence processing (experiment 2). Adopting the aforementioned theories, we hypothesized that if noun and verb categories such as *paint* and *visit* are listed under a single lexical entry, stored in their base category as a noun or verb, respectively, greater selection rates are expected for *the* in *paint* and *to* in *visit* (experiment 1), due to one form being derived from the other, and longer on-line reading times are expected for *to paint* compared to *a paint* and *a visit* compared to *to visit* (experiment 2), as an index of the effect of zero derivation. In contrast, the Dual Entry account, which holds that the two categories of ambiguous words are listed separately in the mental lexicon, predicts no difference in selection rates of *the* and *to* in *paint* and *visit* and no difference in on-line reading times for *a paint* and *to paint*, when frequency and other lexical-semantic features are held equal.

With regard to individuals with post-stroke aphasia, those with fluent aphasia with relatively preserved production of nouns and verbs are expected to show a pattern similar to that of healthy participants. If the two forms of the ambiguous word differ in their underlying morphosyntactic structure and are not simply semantically and phonologically related, then individuals with non-fluent agrammatic aphasia are expected to perform better on ambiguous words in a noun context (*the paint*) than in a verb context (*to paint*). This would suggest that the noun-verb dichotomy in agrammatism is caused by their morphosyntactic complexity rather than lexical-semantics (assuming that the verb form *to paint* necessarily implies the use of an object *a paint).* On the other side, it can be hypothesized that the concept node of “paint” activates the two lemma nodes of *the paint* and *to paint*. If multiple stored representations are activated in parallel, the selection of the most likely representation is governed by lexical factors (e.g., frequency, form typicality, semantic congruency), and thus, individuals with non-fluent agrammatic aphasia would be expected to perform better on the more-frequent forms of these ambiguous words.

## 2. Experiment 1

Experiment 1 consisted of a single-word forced choice phrasal-completion task with categorially unambiguous and ambiguous words, as in (1):

**Table:** 

(1)	A. *Unambiguous noun*: __ *tray (the/to)*
	B. *Unambiguous verb*: __ *eat (the/to)*
	C. *Ambiguous noun*: __ *paint (the/to)*
	D. *Ambiguous verb*: __ *visit (the/to)*

The unambiguous words (A, B) are compatible only with *the* for nouns or *to* for verbs. Accordingly, we expected *the* to be selected for the unambiguous nouns and *to* be selected for the unambiguous verbs. For ambiguous word pairs (e.g., [_N_ *paint*] and [_V_ *visit*]) (C, D), we expected selection rates associated with their base category (i.e., *the* for ambiguous nouns; *to* for ambiguous verbs) if the pairs are related by a derivational process, in keeping with the single entry theory. But, we expected equal selection rates of *the* or *to* if each member of the pair is listed separately within the lexicon, in keeping with the dual entry account. The forced choice phrasal-completion task was selected in order to test individuals with aphasia to covertly produce nouns and verbs, which differentiates this from other studies that mainly used a picture-naming task and required overt derivation and production of nouns and verbs. Importantly, this kind of covert task enables the observation of a possible base-category bias, as is expected for healthy participants, or a lack thereof, as expected for individuals with non-fluent agrammatic aphasia for verbs, but not nouns, without the possible confounds that arise in overt production tasks.

### 2.1. Participants

Participants included 30 right-handed healthy older individuals (17 females; age range = 37–75 years, *M* = 58.53) and 12 individuals with aphasia (5 females; age range = 43–72 years, *M* = 57.67). Participants with aphasia were at least 6 months post-onset of stroke (6–294 months post, *M* = 74.42, *SD* = 81.77). Six of the 12 participants with aphasia showed disproportional deficits in naming verbs compared to nouns as indicated by low scores (less than 85% accuracy) on verb-naming tests of the *Northwestern Assessment of Verbs and Sentences* (NAVS; Thompson, 2011), *Northwestern Naming Battery* (NNB; Thompson and Weintraub, 2014), and *An Object and Action Naming Battery* (OANB; Druks and Masterson, 2000), and noun-verb ratios, obtained from administration of the NNB of > 1.1 (Mack et al., 2015). Importantly, these six participants with aphasia exhibited symptoms consistent with non-fluent agrammatism: low fluency scores on the *Western Aphasia Battery-Revised* (WAB-R; Kertesz, 2007), greater impairment of non-canonical as compared to canonical sentence structures across modalities and greater production impairment of transitive compared to intransitive verbs on the NAVS. In contrast, the other six participants with fluent aphasia showed no verb production impairment as illustrated by high performance (greater than 85% accuracy) on verb-naming tests and no agrammatism across tests. Language testing results and significance for the two groups of participants with aphasia are reported in Table 1. All participants were monolingual English speakers with normal or corrected-to-normal vision and hearing and provided written informed consent, approved by the Institutional Review Board of Northwestern University. Healthy participants had no history of speech-language, learning, or neurological disorders, or psychiatric disturbances (self-reported).

**TABLE 1:** Demographics and language performances for the two groups with aphasia.

	Fluent aphasia	Non-fluent agrammatic aphasia
**Demographic**		
*N*	6	6
Age (number of years)	58.0 ± 12.3	57.3 ± 9.5
Sex, *n* (%) female	2 (33%)	3 (50%)
Education (number of years)	15.0 ± 2.4	15.3 ± 1.9
Post-onset of stroke (number of months)	102.3 ± 106.1	46.50 ± 39.9
**Western aphasia battery**		
Fluency (1–10)	7.5 ± 2.0	3.7 ± 1.5**
Information content (1–10)	8.5 ± 1.2	7.3 ± 1.5
Auditory comprehension (10)	9.6 ± 0.6	7.5 ± 0.5***
Repetition (10)	7.9 ± 1.6	5.5 ± 2.5*
Naming (10)	8.8 ± 0.6	6.8 ± 1.8**
Aphasia quotient (100)	84.6 ± 7.3	62.8 ± 13.9**
**Nouns and verbs naming tests (% correct)**		
OANT object naming	98.5 ± 3.0	60.7 ± 24.4**
OANT action naming	95.5 ± 4.4	52.0 ± 20.7***
NNB noun naming	97.0 ± 5.0	75.0 ± 21.3**
NNB verb naming	98.0 ± 3.1	52.0 ± 21.1***
NNB noun: verb ratio	0.99 ± 0.05	1.53 ± 0.37**
**Northwestern assessment of verbs and sentences (% correct)**		
NAVS verb naming	93.7 ± 6.3	52.6 ± 24.0***
Intransitive (1-arg verbs)	96.7 ± 8.2	60.0 ± 20.0***
Transitive (2 and 3-arg verbs)	93.0 ± 7.0	43.0 ± 26.5**
NAVS verb comprehension	99.2 ± 2.0	98.0 ± 2.7
NAVS argument structure production test	98.0 ± 3.6	67.0 ± 12.6***
Intransitive (1-arg verbs)	100.0 ± 0.0	90.0 ± 11.5
Transitive (2 and 3-arg verbs)	97.3 ± 4.8	62.7 ± 13.3***
NAVS sentence production priming test	64.5 ± 29.4	34.6 ± 16.7
Canonical	81.0 ± 24.9	50.4 ± 19.4
Non-canonical	48.7 ± 37.9	18.6 ± 24.7
NAVS sentence comprehension test	80.5 ± 13.9	53.4 ± 23.2*
Canonical	88.8 ± 12.61	58.6 ± 27.38*
Non-canonical	72.2 ± 16.6	47.8 ± 21.0

Overall accuracy (mean ± standard deviation) for each language test is reported by each group with aphasia. ANOVAs were used to compare groups on all language measures (controlling for language severity as measured by WAB AQ), and Welch two sample t-tests were used to compare groups on demographic variables except for sex (compared using χ^2^ tests). Asterisks indicate significant differences between the two groups with aphasia (*p < 0.05, **p < 0.01, ***p < 0.001).

### 2.2. Materials and procedure

The stimuli consisted of 40 unambiguous and 40 ambiguous nouns and verbs, creating a total of 80 experimental stimuli (see Supplementary Table 1 for a complete list of stimuli). In addition to the 80 experimental stimuli, another 80 words, 40 adjectives, and 40 adverbs paired with either *too/so* or *very/from*, were selected as fillers. Unambiguous stimuli were selected only if the word was used solely as a noun (e.g., *tray*) or as a verb (e.g., *eat*) in the Corpus of Contemporary American English (COCA; Davies, 2008). Ambiguous stimuli (e.g., *paint, visit*) were selected only if the noun and verb forms had: (a) identical orthographic and phonological form (i.e., homographs, homophones), (b) near-equal word frequency of usage as a noun and as a verb (range: 0.85–1.25) and near-equal phrasal frequencies (statistical count of how often *to + verb* vs. *the + noun* appears) based on COCA and (c) specific semantic and syntactic properties characterizing zero-derived words, as discussed above. Given that the directionality of verb-to-noun or noun-to-verb derivation is an unsettled issue in the literature (see Balteiro, 2007; Bram, 2011 for criteria and literature overviews), we adopted both syntactic and semantic analyses to determine the base and derived forms (such as the realization of argument structure, and entailment and semantic regularities).

Verb-to-noun derivations (i.e., derived nouns) in English are generally incompatible with verbal argument structure (AS) and are simple result nominals rather than argument structure nominal (Grimshaw, 1990; Borer, 2013). To test their inability to take arguments, we generated percentages of argument structure of the two forms of ambiguous verbs using the COCA database and applied an AS paraphrase test (Grestenberger and Kastner, 2022). As intended, we found a significant difference between two pairs of words on percentages of AS: [_N_ *visit*] and [_V_ *visit*] [M(SD) = 5.86% (0.07) and 25.80% (0.12), *p* < 0.000]. Crucially, our derived nouns are event nominals, rather than AS nominals, as shown by paraphrase tests which indicate the nouns’ inability to take aspectual modifiers like “for 3 h”: *the visit of the daughter for 3 h versus *they are visiting the daughter for 3 h* (see Supplementary Table 2A for AS of all ambiguous verbs). Moreover, derived nouns cannot accommodate adverbs requiring adjectival modification instead, while the verbal gerund is only compatible with adverbial modification, as shown by paraphrase tests: John’s quick/*quickly visit of the daughter versus John’s quickly/*quick visiting the daughter (Lees, 1960; Chomsky, 1970). Noun-to-verb derivations (derived verbs), on the other side, tend to be transitive (Rimell, 2012) but do not undergo transitive-unaccusative alternation, unlike deadjectival verbs which alternate in transitivity (Hale and Keyser, 1997): We painted the wall/*The wall painted versus *We cleared the screen/The screen cleared* (see Supplementary Table 2B for AS of all ambiguous nouns).

Moreover, from a semantic perspective, we expected there to be a regular relationship between the lexical meaning of the base and derivative. For example, *visit* was classified as a derived *predicate* noun, because the phrase *the visit* denotes an instance or occurrence of the action of visiting [Noun = the act of the Verb (predicate); e.g., “*have a visit*” Marchand, 1969]. Conversely, *paint* was classified as a derived *locatum* verb because it denotes an action that crucially involves a thing like *paint* [Verb = to cause Y to have N in/on it (locatum); e.g., “to color the wall with *paint*” Clark and Clark, 1979]. Therefore, we considered both syntactic and semantic distributions when selecting zero-derived words, and following we will refer to “ambiguous noun” as words that have a noun base category, and “ambiguous verb” as words that have a verb base category.

In addition, the final set of stimuli was selected following the administration of two ranking questionnaires in which a group of healthy native English speakers evaluated the semantic properties of words and as well as phrase acceptability of each noun/verb pair (*n* = 20 and *n* = 11) using a 7-point Likert scale (see Supplementary material for detail descriptions on these questionnaires).

Items in the two ambiguity conditions (unambiguous, ambiguous) were matched for length, orthographic and phonological neighborhood, word and phrasal frequencies, and age of acquisition (all *p*’s > 0.05). Importantly, within each of the two ambiguous conditions, there were no differences between nouns or verbs in their COCA word or phrasal frequencies, length (number of phonemes/morphemes), semantic neighborhoods (all *p*’s > 0.05), or “form typicality” (an estimate of nouniness/verbiness of all of the ambiguous items) as defined by Sharpe and Marantz (2017) (*p* > 0.05). As intended, we also found no significant difference between two pairs of ambiguous words on form frequency: [_N_ *paint*] and [_V_ *paint*] [M (SD) = 4.16 (0.52) and 3.91 (0.56), *p* = 0.154] and [_N_ *visit*] and [_V_ *visit*] [*M* (SD) = 4.39 (0.34) and 4.26 (0.43), *p* = 0.292] or phrasal frequencies: [_N_ *paint*] and [_V_ *paint*] [*M* (SD) = 3.41 (0.56) and 3.52 (0.68), *p* = 0.576] and [_N_ *visit*] and [_V_ *visit*] [*M* (SD) = 3.49 (0.44) and 3.70 (0.45), *p* = 0.139]. However, in the unambiguous conditions, the nouns had a significantly younger age of acquisition and were significantly lower in phrasal frequency than verbs (see Table 2).

**TABLE 2:** The stimuli and statistical results for psycholinguistic variables across conditions.

	Ambiguous		*p* adj.	Unambiguous		*p* adj.
	Nouns (*paint*)	Verbs (*visit*)		Nouns (*tray*)	Verbs (*eat*)	
*N*	20	20		20	20	
# of letters	4.8 (0.9)	5.5 (1.1)	0.288	5.2 (1.1)	6.1 (1.1)	0.030
# of phonemes	3.9 (0.8)	4.5 (1.4)	0.246	4.5 (1.1)	5.2 (1.3)	0.193
# of syllables	1.2 (0.4)	1.6 (0.5)	0.177	1.6 (0.7)	1.9 (0.5)	0.300
# of morphemes	1.0 (0.0)	1.1 (0.2)	0.956	1.1 (0.3)	1.4 (0.5)	0.060
Orthographic neighborhood	7.9 (5.5)	3.8 (5.3)	0.074	5.8 (5.9)	2.1 (4.1)	0.134
Phonological neighborhood	16.1 (13.8)	9.9 (12.9)	0.408	12.8 (15.2)	4.3 (7.4)	0.160
Semantic neighborhood	4517.6 (2983.7)	5502.8 (2401.1)	0.614	1192.8 (2105.8)	3140.7 (2616.0)	0.082
Frequency COCA word (log)	4.2 (0.5)	4.3 (0.4)	0.919	3.9 (0.6)	4.2 (0.5)	0.184
Word frequency (N/V ratio)	4.2:3.9 (1.1)	4.4:4:3 (1.0)	0.680	3.9:1.0 (3.8)	1.7:4.2 (0.4)	<0.001
Frequency COCA phrase (log)	3.4 (0.6)	3.7 (0.5)	0.322	3.1 (0.6)	3.9 (0.5)	<0.001
Age of acquisition (Kuper)	6.1 (1.5)	6.6 (1.8)	0.778	4.9 (0.9)	7.6 (2.0)	<0.001
Form typicality	3.93 (1.99)	4.52 (2.31)	0.389	–	–	

Stimuli consisted of 40 ambiguous and 40 unambiguous words. Values shown are mean (standard deviation). Frequency was extracted from the Corpus of Contemporary American English (COCA; Davies, 2008). Neighborhoods and age of acquisition were extracted from the South Carolina Psycholinguistic Metabase (SCOPE; based on Scott et al., 2019). The measure of “Form Typicality” is based on the model developed by Sharpe and Marantz (2017). A higher value indicates that the form is more typical of a noun than a verb.

**FIGURE 1:**
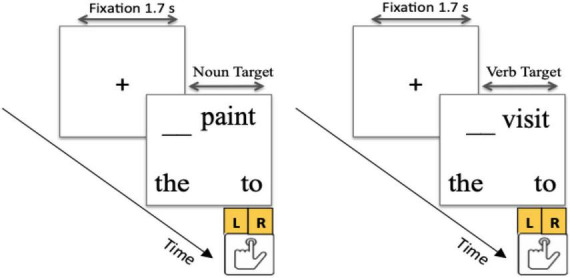
Schematic of ambiguous base-category noun **(left)** and verb **(right)** targets in the forced-choice phrasal-completion task.

All participants performed a forced choice phrasal-completion task. On each trial, a fixation cross was displayed at the center of the computer screen for 1.7 s. Then the target or the filler word was presented, along with two words on the lower left and right bottom of the screen (e.g., *the* and *to*, *too* and *so*, or *very* and *from*). The trial ended with the participant’s button press response (“L” and “R” keys), indicating which of the two words they selected (see Figure 1). The location of two words (e.g., *the* and *to)* was counterbalanced across trials. Detailed instructions and twelve practice trials with feedback were administered prior to the actual experiment for participants to become familiar with the task. The stimulus list was generated randomly for each participant by the program so that the same words were administered in a different random order at each test time. The experiment was presented using E-Prime software (Bates et al., 2012) on a Lenovo desktop computer running Windows XP Professional with an Intel Core 2Quad CPU processor.

### 2.3. Data analyses

Selection Rate (SR) and Reaction Time (RT) were recorded on each trial of the task, with RT measured from the onset of the trial to the subject’s response. Mean SR and RT were calculated for each item and each condition. To check for potential outliers from the RT data, the mean and standard deviation of RTs were calculated for each condition of the task, and any participant with RT above or below three standard deviations of the mean was excluded from the analyses. The RTs were log-transformed, so as to reduce skewness in the distribution. Since the SR was binary (1 = base-compatible response or 0 = base-incompatible response), a standard ANOVA was avoided; instead, a Logistic Mixed Effect Regression analysis was selected. Thus, the analyses were conducted using *logistic regressions (for SRs)* and *linear regressions (for RTs)* with fixed effects for Syntactic Category (nouns vs. verbs) and Ambiguity (unambiguous vs. ambiguous), and Tukey’s honest significant difference (HSD) for the planned *post-hoc* comparisons. The dependent variable was either SR (i.e., proportion of compatible responses: selection of *the* for *tray* and *paint*, and selection of *to* for *eat* and *bite*) for logistic regression or logRT across correct responses for linear regression. For the ambiguous words, both response options (*the* and *to*) were considered “correct” for the purposes of RT analyses. Crucially, the one-sample proportions X-squared test (χ^2^) was used to further analyze SR by determining if the selection of *the* or *to* was significantly different from the selection determined by chance (±50%).

### 2.4. Results

The Selection Rate (SR) and Reaction time (RT and logRT) means and standard deviations for each condition for healthy controls (HC), and participants with fluent aphasia (Aph-fluent) and non-fluent agrammatic aphasia (Aph-nonfluent) are summarized in Table 3. Data from 3 healthy adults were excluded based either on low average SR in the unambiguous conditions (<80%), or on an abnormal RT distribution (3 × mean group SD). One item (*play*) was deleted prior to the analysis because of average low SR (<15%) across participants. Thus, the results are reported below after removing the outliers.

**TABLE 3:** The means and standard deviations of selection Rate (SR) and Reaction Time (logRT and RT) for each condition and each group in Experiment 1.

Group	Condition	SR mean (std.)	logRT mean (std.)	RTmean (std.)
Healthy controls	Unamb nouns	0.99 (0.02)	7.43 (0.35)	1869.5 (710.6)
	Unamb verbs	0.98 (0.03)	7.55 (0.35)	2175.75 (890.9)
	Amb nouns	0.62 (0.19)	7.69 (0.50)	2728.9 (1648.0)
	Amb verbs	0.63 (0.17)	7.66 (0.43)	2595.9 (1401.9)
Aph-fluent	Unamb nouns	0.99 (0.02)	8.18 (0.35)	3968.2 (1242.5)
	Unamb verbs	0.99 (0.02)	8.42 (0.44)	5365.4 (2374.2)
	Amb nouns	0.62 (0.15)	8.51 (0.43)	5986.3 (2475.5)
	Amb verbs	0.61 (0.16)	8.52 (0.34)	6011.4 (1986.26)
Aph-non-fluent	Unamb nouns	0.88 (0.15)	8.60 (0.40)	6436.6 (2905.4)
	Unamb verbs	0.53 (0.24)	8.78 (0.34)	7157.4 (2177.8)
	Amb nouns	0.74 (0.53)	8.81 (0.42)	8126.7 (3583.4)
	Amb verbs	0.48 (0.16)	8.81 (0.42)	7782.5 (3017.6)

*Logistic regression analysis of SR* within each group indicated a significant main effect of Syntactic Category, with SR being significantly higher for nouns over verbs (HC: *b* = −1.51, *SE* = 0.64, *p* = 0.019; Aph-nonfluent: *b* = −2.19, *SE* = 0.36, *p* < 0.000). There was also a significant main effect of Ambiguity (unambiguous vs. ambiguous), with SR being significantly higher in the unambiguous condition compared to the ambiguous condition (HC: *b* = −4.72, *SE* = 0.58, *p* < 0.000; Aph-fluent: *b* = −4.27, *SE* = 1.02, *p* < 0.000; Aph-nonfluent: *b* = −1.15, *SE* = 0.36, *p* = 0.002). Finally, a significant interaction effect between Syntactic Category and Ambiguity was found (HC: *b* = 1.58, *SE* = 0.65, *p* = 0.016; Aph-nonfluent: *b* = 0.93, *SE* = 0.45, *p* = 0.038). However, no main effect of Syntactic Category (*b* = 0.02, *SE* = 1.42, *p* = 0.988) or an interaction effect with Ambiguity (*b* = −0.07, *SE* = 1.45, *p* = 0.959) was found in individuals with fluent aphasia.

Pairwise comparisons revealed that the SR was higher for unambiguous nouns compared to ambiguous nouns (HC: *b* = 4.71, *SE* = 0.58, *p* < 0.000; Aph-fluent: *b* = 4.27, *SE* = 1.03, *p* < 0.000; Aph-non-fluent: *b* = 1.15, *SE* = 0.36, *p* = 0.008), and unambiguous verbs compared to ambiguous verbs (HC: *b* = 3.13, *SE* = 0.29, *p* < 0.000; Aph-fluent: *b* = 4.34, *SE* = 1.02, *p* < 0.000). Importantly, no significant SR differences between unambiguous and ambiguous verbs were found in individuals with nonfluent agrammatic aphasia (*b* = 0.22, *SE* = 0.26, *p* = 0.842).

To assess whether the SR effects could be accounted for by variations of lexical factors and/or language severity, in the follow-up analyses base form frequency or phrasal frequency and WAB-AQ were added to the regression model for *Syntactic Category, Ambiguity*, and their interaction as predictors. Results revealed that all effects persisted, and most of these variables were not significant and did not account for a significant part of the variance in HC (base frequency: *p* = 0.551; phrasal frequency: *p* = 0.018), and Aph-fluent (base frequency: *p* = 0.824, phrasal frequency: *p* = 0.887; WAB -AQ: *p* = 0.370), however, frequency and WAB-AQ accounted for a significant part of the variance only in Aph-non-fluent (base frequency: *p* = 0.006, phrasal frequency: *p* = 0.001; WAB -AQ: *p* = 0.003).

**FIGURE 2:**
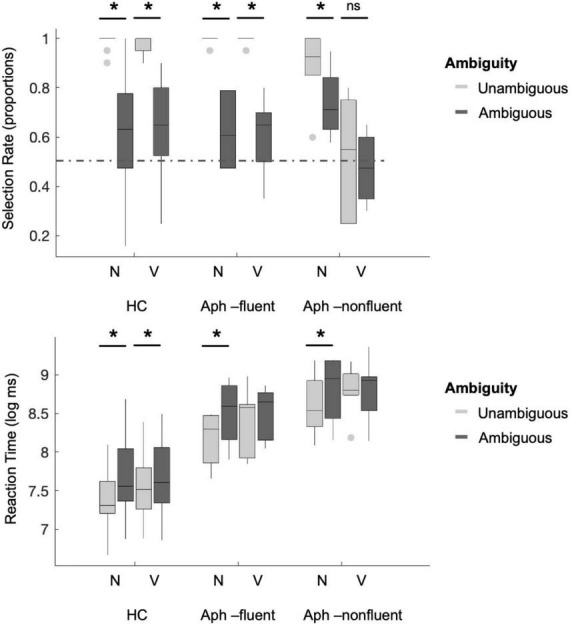
Selection rate and reaction time across conditions and for the three groups. In each panel, the plots are grouped on the x-axis by the syntactic category [Noun (N) or Verb (V)], and grouped by color according to their ambiguity status (unambiguous or ambiguous). Thus, from left to right, the conditions are unambiguous Noun (*tray*), ambiguous noun (*paint*), unambiguous verb (*eat*), and ambiguous verb (*visit*). Selection rate of the and to was significantly different from chance performance for both nouns and verbs conditions, respectively, for healthy controls (HC) and individuals with fluent aphasia (Aph-fluent) but at chance performance for verbs for individuals with non-fluent agrammatic aphasia (Aph-non-fluent) (shown by the dotted line). Reaction time was significantly different between unambiguous and ambiguous conditions but was not different between the two verb conditions only for individuals with aphasia. Asterisks denote significant differences across conditions at *p < 0.001. ns, not significant.

Further, *the one-sample proportions X-squared test* used to test SR against chance performance revealed that SR was significantly above chance for both noun [HC: unambiguous: χ^2^ (1) = 526.09, *p* < 0.000; ambiguous: χ^2^ (1) = 28.07, *p* < 0.000; Aph-fluent: unambiguous: χ^2^ (1) = 114.08, *p* < 0.000; ambiguous: χ^2^ (1) = 6.39, *p* = 0.011] and verb conditions [HC: unambiguous: χ^2^ (1) = 483.56, *p* < 0.000; ambiguous: χ^2^ (1) = 38.93, *p* < 0.000; Aph-fluent: unambiguous: χ^2^ (1) = 114.088, *p* < 0.000; ambiguous: χ^2^ (1) = 5.21, *p* = 0.022]. Importantly, the X-squared test for individuals with non-fluent agrammatic aphasia revealed that SR was significantly above chance for the noun condition [unambiguous: χ^2^ (1) = 69.01, *p* < 0.000; ambiguous: χ^2^ (1) = 24.64, *p* < 0.000], but not for the verb condition [unambiguous: χ^2^ (1) = 0.208, *p* = 0.648; ambiguous: χ^2^ (1) = 0.67, *p* = 0.411]. Figure 2 illustrates SR across conditions for healthy controls, and individuals with fluent and non-fluent agrammatic aphasia.

*Linear regression analysis of logRT* within each group revealed a significant main effect of Syntactic Category (nouns vs. verbs), with responses to verbs being significantly longer than responses to nouns (HC: *b* = 0.14, *SE* = 0.03, *p* < 0.000; Aph-fluent: *b* = 0.27, *SE* = 0.07, *p* < 0.000; Aph-non-fluent: *b* = 0.21, *SE* = 0.08, *p* = 0.013). There was also a significant effect of Ambiguity (unambiguous vs. ambiguous), with significantly longer responses to ambiguous words compared to unambiguous words (HC: *b* = 0.27, *SE* = 0.03, *p* < 0.000; Aph-fluent: *b* = 0.35, *SE* = 0.07, *p* < 0.000; Aph-non-fluent: *b* = 0.27, *SE* = 0.07, *p* < 0.000). Finally, the interaction effect between Syntactic Category and Ambiguity was found (HC: *b* = −0.16, *SE* = 0.05, *p* < 0.000; Aph-fluent: *b* = −0.24, *SE* = 0.10, *p* = 0.016), however, it was not found in individuals with non-fluent aphasia (*b* = −0.20, *SE* = 0.11, *p* = 0.063).

Pairwise comparisons revealed that the RT was higher for ambiguous nouns compared to unambiguous nouns (HC: *b* = −0.27, *SE* = 0.03, *p* < 0.000; Aph-fluent: *b* = −0.35, *SE* = 0.07, *p* < 0.000; Aph-non-fluent: *b* = −0.27, *SE* = 0.07, *p* < 0.001), and ambiguous verbs compared to unambiguous verbs (HC: *b* = −0.12, *SE* = 0.03, *p* = 0.001). However, no significant RT differences between ambiguous and unambiguous verbs were found in individuals with fluent and non-fluent agrammatic aphasia (Aph-fluent: *b* = −0.11, *SE* = 0.07, *p* = 0.421; Aph-non-fluent: *b* = −0.07, *SE* = 0.08, *p* = 0.807).

In addition, to assess whether the RT effects could be accounted for by variations of lexical factors or language severity, in the follow-up analyses base form frequency or phrasal frequency and WAB-AQ were added to the regression model for *Grammatical Category, Ambiguity*, and their interaction as predictors. Results revealed that all effects persisted, and frequency accounted for a significant part of the variance in HC (base frequency: *p* = 0.032; phrasal frequency: *p* = 0.023); however, only WAB-AQ accounted for a significant part of the variance in Aph-non-fluent (base frequency: *p* = 0.066; phrasal frequency: *p* = 0.175; WAB-AQ: *p* < 0.000) and not in Aph-fluent (base frequency: *p* = 0.059, phrasal frequency: *p* = 0.138; WAB-AQ: *p* = 0.558). Figure 2 illustrates RT across conditions for healthy controls, and individuals with fluent and non-fluent agrammatic aphasia.

### 2.5. Discussion

The results of Experiment 1 revealed that healthy participants and individuals with fluent aphasia have a “base-category bias” for ambiguous words, in that they show higher selection rates of *the* for ambiguous nouns (*paint*), and higher selection rates of *to* for ambiguous verbs (*visit*), and likewise for the unambiguous nouns and verbs (*tray* and *eat*). This suggests that individuals are able to access the base category of ambiguous words during lexical access tasks, and are sensitive to their morphosyntactic structure.

Importantly, the two pairs of ambiguous words were matched on form and phrasal frequencies (statistical counts of *to* + verb vs. *the* + noun frequencies). Therefore, the observed base-category bias cannot be caused by the frequency of usage or object vs. event reference. For participants with non-fluent agrammatic aphasia, we found a base-category bias only for nouns (i.e., higher rates of *the* selection for both unambiguous and ambiguous nouns). This finding shows that these participants have a specific deficit in processing verbs relative to nouns, evidenced by the fact that both unambiguous and ambiguous verbs (*eat* and *visit*) were at a chance performance, despite the fact that the ambiguous words have the same phonological and orthographic form as both a noun and a verb. This provides evidence that individuals with non-fluent agrammatic aphasia struggle to process words of a specific syntactic category (in this case, verbs), even those that are equally used as nouns, and even within a covert production task.

However, one limitation regarding these findings is that the task involved a phrasal completion decision over single words, rather than sentences. In natural speech, words appear in phrases and sentences with syntactic scaffolding, thematic information, and prosodic contour that can help to disambiguate the grammatical role of the ambiguous word (e.g., *Anne painted the wall*; *Anne covered the wall with paint*). It could be argued, then, that the observed effect arose because of the artificial nature of the task, and that the deficits for the individuals with non-fluent agrammatic aphasia arose because of task-related constraints rather than the difficulty of accessing a base verb and, in turn, the derived category. Therefore, it is possible that Experiment 1 did not reflect a true effect of complex morphosyntactic structure or on-line structure building and instead arose as a task-related artifact. Experiment 2 addresses this possible confound by evaluating the processing of categorially ambiguous words in more natural sentence contexts.

## 3. Experiment 2

Experiment 2 used an eye-tracking while reading task with the categorially ambiguous nouns and verbs placed in sentence frames that differed only in one disambiguating cue, the presence of *to* or *the*, as in (2). We use “base noun” to refer to those items which have a noun base category and are used as nouns, and “derived noun” to refer to those items which have a verb base category that is zero-derived in order to be used as nouns.

**Table:** 

(2)	A. *Base noun:* Rachel needed ***the** paint* since the house
	looked old.
	B. *Derived verb:* Rachel needed ***to** paint* since the house
	looked old.
	C. *Base verb:* Sarah planned ***to** visit* before the customer
	called.
	D. *Derived noun:* Sarah planned ***the** visit* before the
	customer called.

Eye-tracking while reading is a commonly-used experimental paradigm in psycholinguistics, used to investigate a number of linguistic phenomena. In this paradigm, participants were presented with an entire sentence on a computer screen while their eye movements are recorded by an eye tracker. In this way, eye tracking while reading is somewhat naturalistic–the participants’ eyes are able to move freely throughout the sentence–while also providing fine-grained temporal detail to infer the time-course of processing. The linking hypothesis for this paradigm is that the reader fixates on the object that they are currently attending to, and so a longer fixation time on a given object suggests that it requires more time to process, reflecting on-line cognitive processes (Rayner, 1998). It has been consistently observed that more syntactically complex words or phrases elicit longer reading times (Rayner et al., 1989; Hyönä and Vainio, 2001; Vainio et al., 2003; Staub et al., 2006). In this context, given that the categorially ambiguous words are phonologically identical and have a high degree of conceptual similarity (sharing many parts of their conceptual representation, category-based differences aside), the difference in reading times for (N *paint*) and (V *paint*) should correspond only to differences in the complexity of its morphosyntactic structure.

By comparing condition A and condition B alone, or condition C and condition D alone, the observed effect could be attributed solely to syntactic category differences. Because this experiment shows derivation going in both “directions,” from verb to noun and from noun to verb, we are able to identify the effect of zero-derivation on reading time independent of category effects. If we observe that all of the ambiguous words exhibit faster reading times in the noun context than in the verb context, regardless of base category–in line with the observation that nouns tend to exhibit faster reading times than verbs in general–then we could conclude that zero-derivation does not incur a processing cost, in support of the Dual Entry theory or the single-categorizer approach in Distributed Morphology. However, a systematic reading time slowdown in derived conditions relative to the base conditions for both categories would indicate that zero-derivation does incur a processing cost.

Moreover, it has been observed many times in the literature that verbs take longer to process, across a number of different experimental paradigms (see Vigliocco et al., 2011 for a review). From this, we may conclude that generating a verb (either by derivation or retrieval) involves more processing effort than generating a noun. This may be due to semantic or conceptual factors, where verbs typically denote events that involve several entities that relate to each other in a particular way, or it could be due to morphosyntactic factors, where verbs impose more constraints on other elements in the syntactic structure (through argument structure and selectional restrictions) compared to nouns. Thus, it is not necessarily the case that verbalization (deriving a noun into a verb) will incur the same processing cost as nominalization (deriving a verb into a noun), given that both processes may have different kinds of syntactic and semantic consequences. The key prediction of the zero derivation account is that the effect of derivation should be additive, where the reading time for a derived item {[V (N *paint*)], [N (V *visit*)]} is always longer than that for the base [(N *paint*), (V *visit*)]. The alternate hypothesis, where the processing cost only corresponds to category-based processing differences, would suggest that verbs {[V (N *paint*)], (V *visit*)} are always read slower than their noun counterparts {(N *paint*), [N (V *visit*)]}.

### 3.1. Participants

Fifty-six undergraduates at Northwestern University (college-aged, 18–24 years old) enrolled in an introductory linguistics course participated in the eye tracking while reading study. All were native English speakers and received partial course credit for participating. All had a normal or corrected-to-normal vision.

### 3.2. Materials and procedure

The materials for the eye-tracking study were designed so that the pairs of sentences were unambiguous in their interpretation (the categorially ambiguous word was clearly interpreted as a noun or a verb), but with only minimal differences between the two sentences, so that any observed effects could be attributed only to the difference in morphosyntactic structure. Sentences like those in (2) were developed with completely identical sentential contexts across conditions except for the disambiguating *the* or *to*. As shown in (2), the stimuli include a subject (which was always a recognizable first name, such as *Rachel* or *John*), a verb that could take an infinitive or a noun phrase as its complement (such as *remember*, *expect*, *plan*, or *start*), the disambiguating element (always *the* or *to*), the target item, and a sentence continuation (e.g., a prepositional phrase or a conjunction, designed to provide some context for the main clause of the sentence; the sentence continuation also acts as a “spillover” region, so that any sentence-final reading effects do not interfere with the reading times for the target).

Only a subset of the ambiguous nouns and verbs from Experiment 1 were used in target sentence frames (11 out of 40); several cannot be used intransitively in the verb context, for example, *damage* requires an object, and while *brush* can sometimes appear without an object, in those cases it implies a specific entity as its object (as in *Justin forgot to brush before his dentist appointment*, where *his teeth* would be the implied object). Though verbs like ‘*visit*’ and ‘*paint*’ do suggest that someone or something was visited or painted, the verb does not point to a specific entity as its object unless it is mentioned in the sentence (‘*Sarah planned to visit after the customer called*’ implies that Sarah visited the customer; ‘*Sam needed to paint because the house looked old*’ implies that Sam painted the house)^2^. In order to have a larger set of sentences for the study, the selected ambiguous nouns and verbs appeared in several items. Each word appeared in at least two items (some appeared in up to 6 items), but they never appeared with the same subject, and never with the same preceding verb, in order to reduce any effects that the preceding words might have. In total, there were 24 items, each with four conditions: (A) a base noun used in a noun sentential context, (B) a base noun used in a verb sentential context (derived), (C) a base verb used in a verb sentential context, and (D) a base verb used in a noun sentential context (derived). Because the base nouns and the base verbs were two different sets of words, each item can be thought of as two pairs of sentences, where conditions A and B are tightly matched and conditions C and D are tightly matched, but conditions A and D are not.

Items in the four conditions were matched for length, orthographic and phonological neighborhood, word and phrasal frequencies, form typicality, and age of acquisition (all *p*’s > 0.05). To ensure that there were no significant acceptability differences between conditions, the 96 stimuli (24 items, 4 conditions) were presented in their entirety to 80 native English speakers on Amazon’s Mechanical Turk, who were asked to rate each sentence on a scale of 1 (completely unacceptable) to 7 (completely acceptable). The mean acceptability for each item and condition was calculated for analysis (see Supplementary Table 3 for a complete list of stimuli and Figure 1). Even though there were differences in the distributions of acceptability, they were within a range of reasonable variation.

The stimuli were presented on a computer screen while eye fixations were recorded on an EyeTrack 1000 Plus. Eye gaze was calibrated on a single dimension, with three calibration points, in order to facilitate the calibration process. The gaze was re-calibrated as necessary throughout the experiment. To start the trial, the participants needed to focus their gaze on a small black square on the left side of the screen. Once the eye tracker detected that fixation, the square was replaced with the stimulus. The participants were instructed to read each sentence as naturally as possible. When they were finished, they pressed a button to advance to the next screen and were asked to respond to comprehension questions for half of the sentences at random. The comprehension question always referred to a part of the sentence outside of the critical region (the categorially ambiguous word), and never provided a cue to the category of the critical word. Several practice sentences and their corresponding questions were given at the beginning of the experiment. The items were randomized among a variety of filler types, for a total of 108 items. Each participant was only presented with one condition per item–using (2) as an example, if a participant read the sentence in (2A), “Rachel needed the *paint* since the house looked old,” they would not be presented with any of the other sentences in (2) throughout the experiment, but over the course of the experiment they would see all four conditions, as they appear in other items. The experiment lasted between 30 and 45 min.

### 3.3. Data analyses

To best exhibit the effect of derivation independent of category, this experiment manipulated Syntactic Category of the base (noun vs. verb) and Derivation Status (base vs. derived) as independent factors in a 2 × 2 factorial design. The key comparisons were between base nouns in a noun context and in a verb context (*the paint* and *to paint*) and between base verbs in a noun context and in a verb context (*the visit* and *to visit*). Because the base nouns and base verbs are different lexical items, it is not ideal to compare between the two noun conditions (*the paint* and *the visit*) or the two verb conditions (*to paint* and *to visit*); any observed differences between these could be due to a number of unrelated factors such as phonology, neighborhood effects, conceptual properties, and so on. Derived items provide near-perfect minimal pairs, and by comparing the effect of derivation for both groups of items, it is possible to observe the effect of zero-derivation independent of surface category effects (the category of use; for example, both *to paint* and *to visit* are used as verbs, even though they have different morphosyntactic structures).

The data were cleaned and tidied using packages from the Tidyverse collection in R. Any trials were removed where the critical region was skipped or where there was track loss at the critical region, as well as any outliers greater than 5 standard deviations away from the mean for each condition. There were several participants (*N* = 12) that were excluded because there was track loss at the critical region in more than one-fourth of the trials. Therefore, 44 participants were included in the data analysis below.

While reading, our eyes do not move smoothly along a straight, linear path, but instead jump around in the sentence, going both forward and backward; these jumps are called “saccades.” Each fixation lasts between 200–250 ms. 10–15% of saccades are regressions, or leftward movement, rather than rightward movement (Rayner, 1998). As a result, in eye-tracking studies, there are a number of different measures that can be used for any given region that is being analyzed. The dependent measures included in this analysis were “first fixation,” “first pass,” “regression path,” and “total time.” “First fixation” reading time was measured as the amount of time spent when the gaze first landed in a given region. “First pass” reading time included the first fixation reading time, as well as the time spent at any other point within the region before moving away from it. After moving out of that region to the right, if the gaze regressed back to the given region, the fixation time in the given region during a regression was the “regression path” reading time. The “total time” was the total amount of time that the eyes fixated in that region.

It is generally thought that the “early” measures–the first fixation and first pass reading times–reflect more automatic, low-level processes, while the “late” measures–the regression path reading time–reflect later processes (e.g., integration or reanalysis), but the differences between the effects found in different measures may reflect a number of other factors, such as general oculomotor constraints (Vasishth et al., 2013). For example, an early event could be reflected in late measures if the gaze moves away from the target word to the next region because of a pre-planned eye movement, and thus the effect may only arise when the gaze returns to the target word in the regression path (a late measure). Because of this, we do not have *a priori* hypothesis for which these measures would show an effect of derivation. The null hypothesis for this experiment–that there is no effect of zero-derivation in on-line reading–can be rejected if a significant effect is observed in *at least one* of these measures. Several sets of linear mixed effects regression (LMER) models were fit to the data using the LME4 package in R. For each set of models, one model was fit individually for each reading measure, at the pre-critical region (*Rachel needed to/the…*) and at the critical region (*visit/paint*).

### 3.4. Results

The first set of models predicted log reading time in the pre-critical region with base category and derivation status as fixed effects and random intercepts for subjects. No significant effects were observed in any reading measure for the pre-critical region (*p* > 0.1), as would be expected, because other than *to* and *the*, the text in the pre-critical region was identical (see Table 4).

**TABLE 4:** Results of linear mixed effects regression model for Experiment 2 at the precritical region, with derivation and base category as fixed effects, and subject and item as random effects.

Fixation type	Factor	Estimate (std. error)	Pr (*t*)
First fixation	Intercept	5.264 (0.037)	–
	Derivation	0.054 (0.035)	0.126
	Base	−0.039 (0.035)	0.270
First pass	Intercept	6.031 (0.005)	–
	Derivation	0.006 (0.003)	0.857
	Base	0.028 (0.003)	0.395
Regression path	Intercept	6.046 (0.056)	–
	Derivation	0.011 (0.034)	0.756
	Base	0.030 (0.034)	0.373
Total time	Intercept	6.510 (0.063)	–
	Derivation	0.048 (0.035)	0.162
	Base	0.051 (0.035)	0.139

**FIGURE 3:**
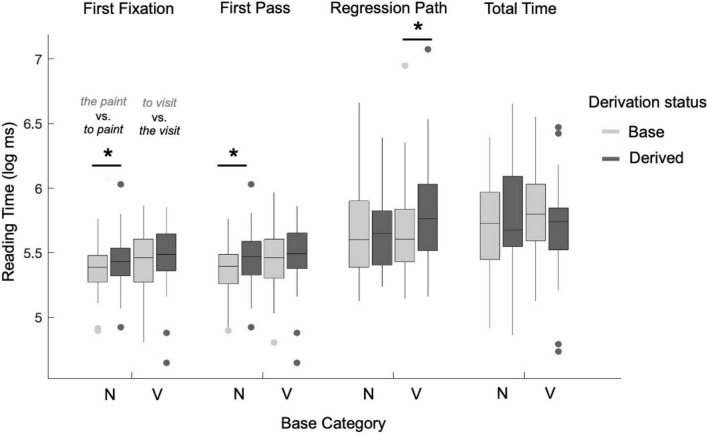
Average log reading time by item (ms) for the categorially ambiguous nouns and verbs in Experiment 2 in four dependent measures (first fixation, first pass, regression path, and total reading time). In each panel, the plots are grouped on the x-axis by the syntactic category of the base [Noun (N) or Verb (V)], and grouped by color according to their derivation status (Base or Derived). Thus, from left to right, the conditions are base noun (*the paint*), derived verb (*to paint*), base verb (*to visit*), and derived noun (*the visit*). A significant effect of derivation (longer reading time) was found for derived verbs relative to the corresponding base nouns in first fixation and first pass reading times, and for derived nouns relative to the corresponding base verbs in regression path reading time (see Table 6). An overall effect of derivation was observed in regression path reading time (see Table 5). Asterisks denote significant reading time differences across conditions at *p < 0.05.

The reading times in the four dependent measures (first fixation, first pass, regression path, and total time) at the critical region are shown in Figure 3. The next set of models predicted log reading time in the critical region (which included only the ambiguous word) by base category, derivation status, and the interaction between them, with random intercepts for subject and item. As shown in Table 5, a significant main effect of derivation was observed in regression path reading time (*p* = 0.027). There were significant main effects of base category in first fixation reading time (*p* = 0.016), first pass reading time (*p* = 0.007), and regression path reading time (*p* = 0.025). The interaction between base category and derivation reached significance in total reading time (*p* = 0.044), and neared significance in first pass reading time (*p* = 0.098). Visual inspection of the data, however, suggested that the effect of derivation arose in the early measures for base nouns, and later measures for base verbs.

**TABLE 5:** Results of linear mixed effects regression model for Experiment 2 at the critical region, with derivation, base category, and the interaction between them as fixed effects, and subject and item as random effects.

Fixation type	Factor	Estimate (std. error)	Pr (*t*)
First fixation	Intercept	5.437 (0.025)	–
	Derivation	0.042 (0.026)	0.108
	Base	0.063 (0.026)	0.016*
	Interaction	−0.078 (0.053)	0.133
First pass	Intercept	5.453 (0.026)	–
	Derivation	0.042 (0.027)	0.125
	Base	0.073 (0.027)	0.007**
	Interaction	–0.090 (0.054)	0.097
Regression path	Intercept	5.716 (0.036)	–
	Derivation	0.090 (0.041)	0.026*
	Base	0.091 (0.041)	0.025*
	Interaction	0.091 (0.081)	0.262
Total time	Intercept	5.748 (0.036)	–
	Derivation	−0.012 (0.041)	0.768
	Base	0.038 (0.041)	0.353
	Interaction	−0.164 (0.081)	0.044*

*p < 0.05, **p < 0.01.

Because the models above grouped the base nouns and base verbs together when estimating the effect of derivation, an additional set of models was developed to tease apart the different effects of derivation for the two groups of words. In these models, log reading time was predicted by derivation nested within levels of base category, again with random intercepts for subject and item. As shown in Table 6, results showed a significant effect of derivation for base nouns in first fixation (*p* = 0.030) and first pass reading time (*p* = 0.026), and a significant effect of derivation for base verbs in regression path reading time (*p* = 0.017).

**TABLE 6:** Results of linear mixed effects regression model for Experiment 2 at the critical region, with a fixed effect of derivation nested within levels of base category, and with subject and item as random effects.

Fixation type	Derivation: base category	Estimate (std. error)	Pr (*t*)
First fixation	Base: noun	5.406 (0.029)	–
	Base: verb	5.469 (0.028)	–
	Derived: noun	0.082 (0.038)	0.030*
	Derived: verb	0.003 (0.037)	0.938
First pass	Base: noun	5.417 (0.030)	–
	Base: verb	5.490 (0.029)	–
	Derived: noun	0.087 (0.039)	0.026*
	Derived: verb	−0.003 (0.038)	0.932
Regression path	Base: noun	5.671 (0.041)	–
	Base: verb	5.761 (0.041)	–
	Derived: noun	0.045 (0.058)	0.441
	Derived: verb	0.136 (0.057)	0.017*
Total time	Base: noun	5.729 (0.042)	–
	Base: verb	5.767 (0.042)	–
	Derived: noun	0.070 (0.058)	0.228
	Derived: verb	−0.094 (0.057)	0.099

*p < 0.05.

*Post-hoc* power analyses, with an effect size of 28 ms (calculated from the difference in means between the derived and base conditions in the raw regression path reading time values by an LMER model), 24 items, and 44 participants, the power level reached about 0.999. These results strongly suggest that zero-derivation does incur a reading time slowdown during on-line sentence comprehension. Additional statistical analyses are included in the Supplementary materials, including models which use the mean acceptability judgment rating as a predictor. Across all of these models, a significant effect of derivation for the derived verbs (*to paint*) arises in the first fixation and first pass reading times, and a significant effect of derivation for the derived nouns (*the visit*) arises in the regression path reading times.

### 3.5. Discussion

To summarize, significant effects of derivation were not found for the pre-critical region, as expected. In the critical region, there was a significant effect of derivation in regression path reading time. When assessing each base category individually, a significant effect of derivation was found for nouns in first fixation and first pass reading time, and for verbs in regression path reading time. Neither base category exhibited a significant effect of derivation in total time. Given the statistical power of this experiment, this finding is likely not due to random error. This suggests that the process of zero-derivation is involved in on-line sentence processing, and gives a clue to the time course that zero-derivation takes during on-line sentence processing. The parser may not immediately recognize a derived word as surprising or costly, but then recognizes the zero morphology during first pass and regression path reading. Once the parser has recognized and built the zero morphology, however, the reading times in that region pattern according to surface category.

Why the effect appeared in two different reading time measures is unclear, though Vasishth et al. (2013) argue that later measures can reflect early events, especially because various oculomotor constraints are not well-understood within the framework of eye tracking while reading. Because a significant effect was observed in several different dependent measures, it is possible to reject the null hypothesis. As discussed earlier, verbalization and nominalization involve different syntactic and semantic consequences, so it is possible that each process would happen on a different time-course, but this would require further experimentation (perhaps with EEG or MEG). Regardless, it is also worth comparing the observed effects with the alternate predictions; if only the category of use had influenced reading times, and given that there is a known effect of syntactic category (where verbs are generally read slower than nouns), we would expect to see opposite effects of derivation for the base nouns and the base verbs–essentially, a derivation “cost” for the base nouns that are derived into verbs [V (N *paint*) -ø], and a derivation “benefit” for the base verbs that are derived into nouns [N (V *visit*) -ø]. Even in first pass reading time, the base verbs show *near equal* reading times with their derived noun counterparts, which suggests that there may be an early category-based advantage that is canceled out by the cost of derivation. Another alternative hypothesis would suggest that only individual lexical items condition reading time (i.e., reading times for *visit* would be the same in any context), but this conclusion would be implausible given that there were significant effects of derivation in several measures.

It has been observed across many psycholinguistic studies that verbs exhibit longer reading times than nouns. This experiment also observes such an effect. However, this is not the focus of the present study; the key comparison is between items related by zero-derivation (*the paint* vs. *to paint*). This experiment observes that these items *do not* pattern solely according to their category of use, but instead that the effect of derivation is additive, regardless of the base category. Furthermore, because “*paint*” and “*visit*” are separate lexical items that are different in many other ways beyond syntactic category (semantic/conceptual representation, phonological form, etc.), we avoid making direct comparisons between *to paint* and *to visit* in order to reduce the influence of potential confounds. Experiment 2 shows that there is an effect of derivation independent of the overall syntactic category effects.

## 4. General discussion

The two storage accounts we have evaluated—single entry and separate entries—make different predictions regarding the processing of categorially ambiguous words. Across the two experiments, the existence of noun/verb base-category biases and longer reading times for derived categories in healthy individuals suggests that some ambiguous words are morphologically complex and subject to morphosyntactic processes where derived forms have no lexical entries distinct from their base forms. Base-category biases appear to be affected in individuals with non-fluent agrammatic aphasia compared to those with fluent aphasia across unambiguous and ambiguous verbs (*to eat/to visit*). That individuals with non-fluent agrammatic aphasia showed a noun bias, but not a verb bias, indicates unimpaired (or normal like) processing of nouns, but selectively impaired verb processing, likely underlying grammatical impairment of this disorder. The study highlights that zero-derivation appears to be affected in post-stroke aphasia (in addition to overt derivations previously reported), especially in cases when individuals with non-fluent agrammatic aphasia had to derive nouns from verbs [N (V *visit*) -ø], stressing the crucial role of the syntactic category of the base (i.e., verbs) in performing morphological processes. These findings provide insights into the representation of categorially ambiguous words, extending our knowledge of these words in normal and impaired lexical processing.

### 4.1. Category bias

The results of Experiment 1 indicated base-category bias effects across syntactic categories: healthy adults showed greater selection rates of *the* for *tray* and *paint* and *to* for *eat* and *visit.* These results show that there is systematic agreement across participants as to which form of the categorially ambiguous word is the base category. This supports the single-entry hypothesis in that one must process the base category before accessing the derived category, as well as a Distributed Morphology approach which allows for multiple categorizers. In terms of processing cost, a verb disadvantage was found across ambiguity conditions, which substantiates previous studies showing longer response times for verbs (actions) compared to nouns (objects) (e.g., Sereno, 1999; Tyler et al., 2001; Bogka et al., 2003; Druks et al., 2006). An additional finding, the ambiguity disadvantage effect, could be due to either category ambiguity and response competition or accessing the derived category. For the critical ambiguous words, both response options (the two meanings of the noun-verb homonyms) are available and compete for selection, leading to increased use of computational resources (see Beretta et al., 2005; Eddington and Tokowicz, 2015). On the other hand, the observed ambiguity effect may also reflect the cost of deriving a more complex structure, given that the ambiguous word would be compatible with the base and derived structure (unlike “paper” which is ambiguous just in meaning), and thus might incur additional processing costs (Lukic et al., 2019).

Individuals with aphasia, interestingly, showed two distinct patterns: (1) a noun/verb base-category bias similar to that in healthy participants for those with fluent aphasia, and (2) a noun bias, but not a verb bias, across the two ambiguity conditions for individuals with non-fluent agrammatic aphasia. These results suggest that syntactic categories may be more vulnerable to breakdown in base verbs than in base nouns in grammatically-impaired individuals with non-fluent aphasia. Extending, previous limited findings on retrieval of noun-verb homonyms in aphasia (e.g., Caramazza and Hillis, 1991; Goldberg and Goldfarb, 2005), the current experiment systematically varied stimuli by differentiating noun-based and verb-based categories of the ambiguous words. On some accounts, the representation of some ambiguous nouns and verbs contained both the base and derived forms, and for that reason, such representations could be affected by a selective base impairment. Thus, it is possible that impaired access to the base category (i.e., verbs, as in *to visit*) prevents retrieval of the derived category (i.e., noun, as in *the visit*) which thus impacts performance in the grammaticality decision task for our individuals with non-fluent agrammatic aphasia.

Furthermore, a bulk of research regarding lexical access of complex words in aphasia has observed that people with agrammatic aphasia have difficulties in producing deverbal items such as (overtly) derived nouns from verbs (e.g., Luzzatti and De Bleser, 1996; Marangolo et al., 2003; Manouilidou et al., 2021). A recent lexical-decision study by Manouilidou and colleagues revealed that both individuals with stroke-induced agrammatic aphasia and those with agrammatic variants of primary progressive aphasia have difficulties processing derived pseudowords and more specifically fail to detect violations in deverbal word formation (*reheavy and *reswim). In the current study, the distinct patterns of selection rates associated with the syntactic category of the base were observed—while individuals with fluent aphasia show an “ease” access to base across syntactic categories, individuals with non-fluent agrammatic aphasia clearly had difficulties with the verb base during covert production even after controlling the two pairs of ambiguous words for lexical variables such as form or phrasal frequency, and form typicality. However, one might argue that individuals with non-fluent agrammatic aphasia showed a lack of verb bias due to difficulty processing the grammatical morpheme “to” compared to the article “the.” This is certainly an important point, nonetheless, it’s unlikely given their unimpaired production of non-finite verb forms (e.g., *The boy likes to eat the hamburger*; see Lee and Thompson, 2017).

The source of the category deficits in non-fluent agrammatic aphasia still is not entirely clear, and merits further investigation. Syntactic category effects may be attributed to either semantic properties (difficulties with object or action representations) or morpho-syntactic processes, including impaired encoding of lexical features (noun/verb distinction), derivation of the complex morphosyntactic structure, an impairment in the rule-governed process for verbs (*to + V*), or an interaction between any of these components. However, this study does shed some light on the representations of these categorially ambiguous items. Although the participants had preserved conceptual knowledge of the verbs as shown in standardized tests (see Table 1), these patients selected *to* and *the* at chance for ambiguous verbs. If the ambiguous pairs were only related by homophony, and not related by derivation, we would expect to see participants with non-fluent agrammatic aphasia selecting *the* more often for the ambiguous verbs (*visit*), knowing that the participants with non-fluent aphasia struggle to produce verbs in general. Instead, because the participants select *the* or *to* at chance, this may suggest that these items are derivationally related, and that accessing the derived category necessarily involves first accessing the base category. This illustrates that the representation of syntactic category is more complex than just the grammatical or semantic function of a word, and thus the deficits related to syntactic category may arise as the result of the interaction of several processes.

### 4.2. Processing cost of zero morphology

The results of Experiment 2 corroborated the findings from Experiment 1, indicating that people are sensitive to the base category of a word in on-line sentence processing, even if that word can be used in multiple syntactic categories. This suggests that implicit knowledge of a word’s base category and possible derived categories must be available for lexical processing during sentence computation. Furthermore, the results indicate that the processes of deriving a verb from a noun and deriving a noun from a verb are both costly–perhaps in different ways. This experiment showed a systematic effect of zero-derivation in a sentential context like that observed for single-word lexical access, showing that the effect observed in Experiment 1 is robust even when the ambiguous word is integrated into an unambiguous structure. Based on this evidence, we conclude that the observed effects are related to the syntactic complexity of the ambiguous words, and not due to lexical effects or task-related artifacts.

These findings have significant implications for a theory of word recognition. Firstly, words like (N *paint*) and (V *paint*) should not be treated as lexical items solely related by phonology and semantic similarity. These results would be challenging to explain for the Interactive Activation Model (McClelland and Rumelhart, 1981) and other connectionist models of word retrieval where distributed lexical representations are instantiated as unique patterns of activation that compete for selection. Those models would predict that the multiple candidates of a categorially ambiguous word compete until one “winner” is selected, suggesting that there would be a slower response for both forms of the ambiguous word compared to a word with only one candidate form. Alternatively, they might predict that syntactic context would inhibit the incorrect category and facilitate the activation of the correct one, and thus there would be no reading time slowdown for either form of the word. These results do not follow either of those patterns.

These findings are also inconsistent with any model of language processing that treats these categorially ambiguous words as a simple semantic ambiguity or polysemy (like *bat* or *paper*). The “Context Dependent” model of lexical ambiguity (Schvaneveldt et al., 1976; Simpson, 1981) argues that the discourse or sentence context would inhibit the meaning that is not relevant to the sentence (this is complicated for words like *paint* where there seems to be an entailment relationship between [_N_ *paint*] and [_V_ *paint*]). Such models would not predict a slower reading time for either form. The “Ordered Access” model (Hogaboam and Perfetti, 1975; Forster and Bednall, 1976) suggests that different interpretations are retrieved in order based on their frequency of usage. However, the items in Experiments 1 and 2 were balanced on frequency; unless the derived category was consistently less frequent than the base category for every ambiguous word, this model would not be able to explain these results. Finally, the “Exhaustive Access” model (Killion, 1979; Onifer and Swinney, 1981) argues that all possible meanings would be activated, and the context would help to determine which inappropriate interpretations should be suppressed. Again, this kind of model would predict that both categories would induce a slower reading time compared to unambiguous words, corresponding to the decision time, but would not predict one form to be read slower than another. None of these models of lexical ambiguity would be able to predict the systematic reading time slowdown for the derived category relative to the base category.

### 4.3. Implications for the organization of the lexicon

The ambiguous noun/verb pairs employed in the current study represent an interesting group of homonyms because of their potential to further our understanding of the mental lexicon. Firstly, these results provide evidence that categorially ambiguous words must be related by a process of zero-derivation. The word’s form is compatible with both the base and derived structures, creating ambiguity in the derivational status of words such as *paint* and *visit*. In a Dual Entry approach, words like (N *paint*) and (V *paint*) would be represented as separate lexical items that happen to share a high degree of semantic and phonological similarity. However, the results of both Experiment 1 and Experiment 2 suggest that a word like *paint* is stored as a noun, and that (V *paint*) must be derived from (N *paint*) through a grammatical process. Experiment 2 especially shows that this effect is more than just a lexical or conceptual bias for the base category, but that there is a cost of derivation related to morphosyntactic structure building. This conclusion would be challenging for theories of the lexicon where lexical items cannot be syntactically complex, or where the ambiguous words would be represented as different words stored in two different lexical entries (Jackendoff, 1975; Aronoff, 1976; Di Sciullo and Williams, 1987).

This would also be challenging for theories where a word like *paint* only receives its category based on its usage, or theories where it would be represented as a fully abstract “root” that has no category until it enters into a syntactic structure that involves a single “categorizer,” such as in some approaches to Distributed Morphology (Halle and Marantz, 1994; Gaston and Marantz, 2018). Other approaches to Distributed Morphology assume, however, that a single root can receive multiple categorizers stacked on top of each other, as discussed by Harley (2009); in order for this to account for the results of Experiments 1 and 2, it must be the case that the root always attaches first to one categorizer, and then other affixes or categorizers. For example, the root for *paint* would need to first attach to a noun categorizer before attaching to the verb categorizer in order to derive (V *paint*). There is good evidence that roots are not specified for syntactic category, but the implication here is that the semantic representation (where the base category is implied in the derived category) is isomorphic with the morphosyntactic structure, suggesting a highly systematic relationship between semantics and syntax. As long as this assumption is met, these data can also be explained by other non-lexicalist theories of syntax, such as the non-semiotic approach (Preminger, 2021) or Nanosyntax (Starke, 2009).

### 4.4. Limitations and future directions

Because the current study is a relatively novel investigation into the status of zero-derivation in both normal and impaired lexical processing, there are several expected limitations. While individuals with non-fluent agrammatic aphasia had clear difficulties with verb retrieval and lack of a verb bias, individuals with fluent aphasia did not show the reverse patterns of noun retrieval impairments and a corresponding lack of a noun bias. This is because our 6 patients with fluent aphasia were mild and showed overall high performances on standardized tests, so the lack of power prevented such comparisons. Future research that identifies biases in both directions will strengthen our conclusions and generalize our results. Furthermore, if implicit (zero) morphology causes difficulty in retrieving nouns and verbs (as shown in *visit*), then the same argument could be made for explicit morphology. The performance of individuals with aphasia on morphologically complex items such as gerunds (*paint ∼ painting*), deadjectival verbs (*beauty* ∼ *beautify*), and deverbal adjectives (*amaze ∼ amazing*) could also be assessed in future studies. These studies of word derivation will add to the growing body of research supporting the existence of morphologically structured complex lexical representations, and contribute to a better understanding of how those representations are disrupted in language disorders. Another important area of future research would be to understand the time course of word-derivation in agrammatism and anomia, and examine the recovery patterns [e.g., effects of treatment focused on one category (e.g., Verb) on generalized improvement to another category (e.g., Noun) in individuals with aphasia].

Another aspect to investigate is the comparison between different kinds of zero-derived nouns and their available interpretations. Iordăchioaia et al. (2020) developed a database of 1,000 zero-derived nouns, and categorized them based on the lexical semantics of their base verbs and the interpretations they may receive (e.g., event, result state, agent). Though our study focused on event nominals, rather than AS nominals, it is possible that we would see different effects for these groups of items depending on their semantic and syntactic representations.

Moreover, future studies should investigate processing differences in verbalization versus nominalization. Experiment 2 exhibited significant effects for both verbalization and nominalization, but in different reading measures; verbalization was shown to be significant in early measures (first fixation and first pass), while nominalization was only shown to be significant in the regression path. As discussed above, verbalization is associated with event structure that relates entities in a particular way, and also imposes constraints on the other elements of the sentence through argument structure and selectional restrictions; although nominalization may be involved in some of these things as well, it is greatly reduced compared to verbs, instead just generating an entity. As a result, it is possible that there would be different time-courses for the two processes in on-line production or comprehension. This experiment cannot address this question, given the constraints on the analysis of eye-tracking measures described in Section “3.5. Discussion,” but we hope that future work will shed some light on this issue, perhaps using electroencephalography (EEG) or magnetoencephalography (MEG) to get more fine-grained temporal measures of the neural processes involved in nominalization and verbalization.

## 5. Conclusion

This study presents two experiments showing that categorially ambiguous words such as *paint* and *visit* are related by the word-formation process of conversion or zero-derivation, and not simply phonological and semantic similarity. Importantly, healthy controls and individuals with fluent aphasia show base-category biases across syntactic categories, whereas those with verb deficits show a syndrome-specific deficit—that is, no verb bias consistent with impaired retrieval of the derived category (i.e., noun). Experiment 2, furthermore, shows that when the categorially ambiguous words are placed into an unambiguous sentence context, the derived form exhibits longer reading times than the base form, independent of syntactic category.

Taken together with past findings on lexical processing, the present findings cannot be accounted for by lexical frequencies or top-down contextual information but rather suggest that language users have implicit knowledge of a word’s base category that is available during on-line lexical access processes. The zero morphology can be identified by the parser, and incurs a processing cost related to the morphosyntactic complexity of the derived form. Although at this time we do not have clear hypotheses about the nature of the brain mechanisms that compute this grammatical process of zero morphology and/or neural bases underlying the organization of complex lexical representations, it is clear that category ambiguity may be more vulnerable to breakdown in individuals with non-fluent agrammatic aphasia than in those with fluent aphasia. These findings present a clear challenge to models of the lexicon where lexical items cannot be syntactically complex, as well as models that would treat these cases of category ambiguity as polysemy or homophony (like *paper* or *bat*).

## Data availability statement

The datasets presented in this study can be found in online repositories. The names of the repository/repositories and accession number(s) can be found below: Anonymized grammaticality judgment data for Experiment 1 and eye-tracking data and acceptability judgments for Experiment 2 are available at https://github.com/alex-krauska/Zero-Morphology.

## Ethics statement

The studies involving human participants were reviewed and approved by the Institutional Review Board of Northwestern University. The patients/participants provided their written informed consent to participate in this study.

## Author contributions

SL and AK supported conceptualization, data acquisition, data analyses, interpretation of the data, and writing and revising the original draft. SL, AK, and CKT provided analytical support and secured funding. MY and CKT provided supervision, conceptualization, data interpretation, writing, and editing. All authors contributed to the article and approved the submitted version.

## Footnotes

1 In the theoretical literature, some researchers refer to this process as conversion, whereas others refer to it as (zero-) derivation. This distinction is irrelevant in the current paper, and we used the term “morphological process” to refer to conversion/derivation terms interchangeably.

2 Because the effect of derivation was observed not just for zero-derived verbs, but also for zero-derived nouns, and that both base verbs (*to visit*) and derived verbs (*to paint*) would likely imply an object in a similar way, it is unlikely that this effect is driven solely by the implication of an object (though it could contribute to the different effect sizes for the two groups of items if verbs in one of the two groups are more inclined to do so).
