# Supplementary material for: The role of category ambiguity in normal and impaired lexical processing: can you paint without the paint?
Source: Front Hum Neurosci. 2023 May 4;17:1028378. doi: 10.3389/fnhum.2023.1028378 (PMC10192584; doi:10.3389/fnhum.2023.1028378)
Supplement: Supplementary file 1 [file Data_Sheet_1.docx]

***Supplementary Material***

# Supplementary Data

**Experiment 1 Stimuli rating questionnaires**

*Semantic relatedness questionnaire*

To evaluate the semantic properties of *ambiguous nouns and verbs*, *twenty right-handed healthy participants* (17 females, 28 ± 4.9 years old, 18.3 ± 1.95 years of education) were presented with noun and verb pairs (e.g., a fight/to fight) and were instructed to rate the degree of semantic similarity between the meaning of the noun and the verb form using a 7-point scale (1 = low similarity, 7 = high similarity). Two practice items were given in order to instruct participants to distinguish meanings between similar and dissimilar pairs. For each of these two practice items participants were given a short scenario to read and then they were presented with two sentences. The target item was used as a noun in one sentence and as a verb in the other. For example, the first practice item fight (for which the verb and noun forms have similar meanings) was tested by presenting the following scenario: “John watched the hockey game.” Then two sentences were presented: one with the target word used as a noun (“One of the players started a fight during the game”) and one with the target word used as a verb (“One of the players started to fight with his opponent”). Similarly, to test the second practice item phone (for which the verb and noun forms have dissimilar meanings), the scenario “John needs to see Mary.” preceded the presentation of sentences with the target word used either as a noun (“John gave a ring to Mary.’) or as a verb (“John needs to ring the bell.”). Used as a noun phrase, the word ring is interpreted as a physical object, whereas when entered into a verb phrase it is interpreted as an action, i.e., making a ring call.

*Acceptability judgment questionnaire*

In order to obtain the acceptability of *ambiguous noun/verb word pairs*, the target words were presented in minimal phrasal contexts (i.e., the fight and to fight) without a sentence, and mean rankings were calculated for each noun and verb pair. We asked *eleven right-handed healthy participants* (7 females, 28 ± 3.4 years old, 16.7 ± 1.4 years of education) to rate nouns and verbs preceded by syntactic context (the/to fight) on a 1-7 scale (1 = low acceptability, 7 = high acceptability): *“The words you will see may refer to either objects or actions. Each word may appear both in a noun and in a verb phrase. Rate how acceptable each phrase is by assigning a digit between 1-totally unacceptable and 7- totally acceptable. Totally acceptable means that a native English speaker is likely to produce it, whereas totally unacceptable means that a native English speaker would not produce it. Please try to use THE FULL SCALE OF VALUES (ranging from 1 to 7), where 1 corresponds to totally unacceptable and 7 indicates totally acceptable phrases.”*

Based on the results of the two questionnaires, high-acceptability noun and verb pairs were divided into two groups – those with similar (mean ratings > 4.0) and those with dissimilar meaning (mean ratings < 4.0). Accordingly, noun/verb pairs with highest rated semantic similarity (such as ‘the/to fight’), and lowest rated noun/verb pairs (such as ‘the/to phone’) were selected for eligibility in the experiment 1.

# SUPPLEMENTARY TABLE 1:

|  | **TABLE 1: Experiment 1 (a forced-choice phrasal-completion task) Stimuli** | | | | | | | | |
| --- | --- | --- | --- | --- | --- | --- | --- | --- | --- |
|  |  |  | **COCA Word log Frequency (mean)** | | **COCA Phrase log Frequency (mean)** | | **Semantic Relatedness (mean (sd))** | **Acceptability Judgment (mean (sd))** | |
| ***No*** | ***Item*** | ***Condition*** | ***N*** | ***V*** | ***the N*** | ***to V*** | ***a N vs. to V*** | ***the N*** | ***to V*** |
| 1 | brush | AmbN | 4.08 | 3.87 | 3.4 | 3.22 | 5.4 (1.9) | 7 (0) | 7 (0) |
| 2 | buckle | AmbN | 3.18 | 3.26 | 2.37 | 2.7 | 5.1 (2.17) | 7 (0) | 6.64 (1.21) |
| 3 | comb | AmbN | 3.31 | 3.13 | 2.58 | 2.62 | 5.35 (2.13) | 7 (0) | 6.82 (0.40) |
| 4 | cover | AmbN | 4.65 | 4.78 | 4.03 | 4.39 | 4.75 (2.1) | 7 (0) | 6.91 (0.30) |
| 5 | crack | AmbN | 4.13 | 3.99 | 3.41 | 3.6 | 4.5 (2.12) | 6.91 (0.30) | 6.64 (1.21) |
| 6 | drill | AmbN | 3.91 | 3.63 | 3.37 | 3.18 | 4.8 (2.44) | 6.91 (0.30) | 6.91 (0.30) |
| 7 | filter | AmbN | 3.97 | 3.38 | 3.15 | 3.05 | 5.6 (1.64) | 7 (0) | 7 (0) |
| 8 | hook | AmbN | 4.17 | 3.7 | 3.75 | 3.23 | 4.85 (1.95) | 6.91 (0.30) | 6.82 (0.60) |
| 9 | iron | AmbN | 4.48 | 3.17 | 3.72 | 2.8 | 4.5 (2.33) | 7 (0) | 6.91 (0.30) |
| 10 | label | AmbN | 4.22 | 3.68 | 3.69 | 3.19 | 5.4 (1.82) | 6.91 (0.30) | 7 (0) |
| 11 | lock | AmbN | 4.13 | 4.05 | 3.52 | 3.54 | 5.75 (1.55) | 6.64 (1.21) | 7 (0) |
| 12 | mark | AmbN | 4.98 | 4.18 | 3.77 | 3.7 | 4.35 (2.28) | 7 (0) | 7 (0) |
| 13 | match | AmbN | 4.42 | 4.38 | 3.57 | 3.96 | 1.95 (1.67) | 7 (0) | 7 (0) |
| 14 | paint | AmbN | 4.33 | 4.22 | 3.61 | 3.81 | 5.6 (1.7) | 6.73 (0.90) | 7 (0) |
| 15 | play | AmbN | 4.93 | 5.3 | 4.12 | 4.91 | 2.15 (1.63) | 6.45 (1.51) | 6.64 (0.92) |
| 16 | recruit | AmbN | 3.32 | 3.78 | 2.13 | 3.56 | 4.7 (2.32) | 6.27 (1.85) | 6.91 (0.30) |
| 17 | seal | AmbN | 3.99 | 3.71 | 3.25 | 3.27 | 2.75 (2.24) | 6.64 (1.21) | 7 (0) |
| 18 | stamp | AmbN | 3.8 | 3.23 | 2.86 | 2.81 | 4.65 (2.18) | 7 (0) | 6.91 (0.30) |
| 19 | switch | AmbN | 4.12 | 4.17 | 3.59 | 3.71 | 3.85 (2.11) | 6.82 (0.60) | 6.73 (0.90) |
| 20 | test | AmbN | 5.02 | 4.53 | 4.29 | 5.17 | 5.3 (1.92) | 6.84 (0.46) | 6.87 (0.42) |
| 21 | answer | AmbV | 4.97 | 4.82 | 3.23 | 3.75 | 5.9 (1.33) | 7 (0) | 7 (0) |
| 22 | attack | AmbV | 4.9 | 4.4 | 4.18 | 4.03 | 5.75 (1.59) | 7 (0) | 6.82 (0.60) |
| 23 | blame | AmbV | 4.09 | 4.55 | 3.76 | 4.11 | 6.4 (1.5) | 6.45 (1.81) | 6.91 (0.30) |
| 24 | chase | AmbV | 4.18 | 3.96 | 3.43 | 3.43 | 5.95 (1.64) | 7 (0) | 7 (0) |
| 25 | claim | AmbV | 4.59 | 4.57 | 3.83 | 3.91 | 5.5 (1.93) | 7 (0) | 7 (0) |
| 26 | damage | AmbV | 4.71 | 3.91 | 4.04 | 3.11 | 6.25 (1.45) | 6.91 (0.30) | 6.91 (0.30) |
| 27 | hug | AmbV | 3.88 | 3.71 | 2.42 | 3.2 | 6.15 (1.39) | 6.91 (0.30) | 7 (0) |
| 28 | kick | AmbV | 4.04 | 4.34 | 2.88 | 3.83 | 5.85 (1.39) | 6.36 (0.92) | 6.82 (0.60) |
| 29 | kiss | AmbV | 4.18 | 4.26 | 3.23 | 3.75 | 6.2 (1.51) | 6.73 (0.90) | 7 (0) |
| 30 | limit | AmbV | 4.33 | 4.26 | 3.58 | 3.88 | 5.3 (1.69) | 7 (0) | 7 (0) |
| 31 | load | AmbV | 4.25 | 3.89 | 3.39 | 3.38 | 5 (1.92) | 6.82 (0.60) | 6.82 (0.60) |
| 32 | offer | AmbV | 4.41 | 4.93 | 3.62 | 4.43 | 5.75 (1.68) | 6.84 (0.46) | 6.87 (0.42) |
| 33 | praise | AmbV | 4.02 | 3.84 | 2.94 | 3.13 | 6.2 (1.15) | 6.91 (0.30) | 6.91 (0.30) |
| 34 | promise | AmbV | 4.48 | 4.51 | 3.89 | 3.23 | 5.6 (1.96) | 7 (0) | 6.91 (0.30) |
| 35 | protest | AmbV | 4.21 | 3.89 | 3.29 | 3.65 | 5.8 (1.36) | 6.73 (0.65) | 6.64 (0.92) |
| 36 | request | AmbV | 4.5 | 3.95 | 3.73 | 3.39 | 6 (1.49) | 6.91 (0.30) | 6.45 (1.81) |
| 37 | rescue | AmbV | 4.2 | 4 | 3.62 | 3.68 | 5.7 (2.13) | 6.45 (1.51) | 6.91 (0.30) |
| 38 | reward | AmbV | 4.12 | 3.6 | 3.34 | 3.21 | 6 (1.65) | 7 (0) | 6.45 (1.81) |
| 39 | support | AmbV | 5.17 | 5.06 | 4.08 | 4.65 | 5.45 (1.61) | 6.45 (1.21) | 6.73 (0.90) |
| 40 | visit | AmbV | 4.57 | 4.73 | 3.36 | 4.34 | 6 (1.41) | 6.91 (0.30) | 6.82 (0.60) |
| 41 | barn | UnambN | 4.07 | N/A | 3.72 | 1.28 |  |  |  |
| 42 | bell | UnambN | 4.54 | N/A | 3.77 | 2.21 |  |  |  |
| 43 | bucket | UnambN | 3.99 | 1.04 | 3.37 | 1.18 |  |  |  |
| 44 | celery | UnambN | 3.59 | N/A | 2.27 | 0.95 |  |  |  |
| 45 | deer | UnambN | 4.29 | N/A | 3.49 | 2.26 |  |  |  |
| 46 | desk | UnambN | 4.63 | N/A | 3.93 | 1.79 |  |  |  |
| 47 | frog | UnambN | 3.79 | N/A | 3.18 | 1.53 |  |  |  |
| 48 | grape | UnambN | 3.64 | N/A | 2.73 | 1.23 |  |  |  |
| 49 | guitar | UnambN | 4.26 | N/A | 3.45 | 1.68 |  |  |  |
| 50 | helmet | UnambN | 4.01 | N/A | 3.2 | 1.73 |  |  |  |
| 51 | jacket | UnambN | 4.4 | N/A | 3.3 | 0.6 |  |  |  |
| 52 | lemon | UnambN | 4.24 | N/A | 3.08 | 1.59 |  |  |  |
| 53 | lid | UnambN | 3.91 | N/A | 3.6 | 0.7 |  |  |  |
| 54 | pillow | UnambN | 3.98 | N/A | 3.35 | 1.15 |  |  |  |
| 55 | sandal | UnambN | 2.79 | N/A | 1.45 | 0.95 |  |  |  |
| 56 | shirt | UnambN | 4.57 | N/A | 3.42 | 1.2 |  |  |  |
| 57 | stapler | UnambN | 2.52 | N/A | 1.8 | 0.04 |  |  |  |
| 58 | tray | UnambN | 3.92 | N/A | 3.24 | 1.08 |  |  |  |
| 59 | wallet | UnambN | 4.02 | N/A | 2.97 | 0.78 |  |  |  |
| 60 | zebra | UnambN | 3.21 | N/A | 2.5 | 1.2 |  |  |  |
| 61 | adopt | UnambV | 1.04 | 4.2 | 0.7 | 3.7 |  |  |  |
| 62 | carve | UnambV | 1.97 | 3.54 | 1.18 | 3.21 |  |  |  |
| 63 | deliver | UnambV | N/A | 4.48 | 1 | 4.15 |  |  |  |
| 64 | destroy | UnambV | N/A | 4.5 | 1.3 | 4.14 |  |  |  |
| 65 | detect | UnambV | N/A | 4.11 | 0.48 | 3.84 |  |  |  |
| 66 | discuss | UnambV | N/A | 4.68 | 0.7 | 4.39 |  |  |  |
| 67 | eat | UnambV | 1.58 | 5.05 | 2.08 | 4.58 |  |  |  |
| 68 | erase | UnambV | N/A | 3.68 | 1.62 | 3.27 |  |  |  |
| 69 | explore | UnambV | N/A | 4.45 | 1.52 | 4.2 |  |  |  |
| 70 | follow | UnambV | 3.13 | 5.02 | 2.49 | 4.51 |  |  |  |
| 71 | imitate | UnambV | N/A | 3.44 | 0 | 3.18 |  |  |  |
| 72 | inspect | UnambV | N/A | 3.59 | 0.6 | 3.33 |  |  |  |
| 73 | invent | UnambV | N/A | 3.67 | 0 | 3.24 |  |  |  |
| 74 | learn | UnambV | 1.72 | 5.16 | 1.72 | 4.77 |  |  |  |
| 75 | locate | UnambV | N/A | 4.01 | 0.95 | 3.78 |  |  |  |
| 76 | observe | UnambV | N/A | 4.2 | 0.7 | 3.82 |  |  |  |
| 77 | promote | UnambV | N/A | 4.51 | 0.6 | 4.27 |  |  |  |
| 78 | propose | UnambV | N/A | 4.03 | 0.7 | 3.46 |  |  |  |
| 79 | pursue | UnambV | N/A | 4.33 | 0.85 | 4.12 |  |  |  |
| 80 | resist | UnambV | 1.04 | 4.21 | 1.86 | 3.79 |  |  |  |

SUPPLEMENTARY TABLE 2:

|  | **TABLE 2A: Experiment 1 Ambiguous Verb-base Stimuli (derived nouns)** | | | | | |
| --- | --- | --- | --- | --- | --- | --- |
|  |  |  | **Argument Structure COCA**  **frequency (mean)** | | **Argument Structure Acceptability Judgment** | |
| ***No*** | ***Item*** | ***Condition*** | ***# of "the __ of * NOUN" uses*** | ***# of "to ___ * NOUN" uses*** | ***"the ___ of ____ for three hours"*** | ***"__ing the __ for three hours"*** |
| 21 | answer | AmbV | 0.13% | 37.34% | The answer of the question for three hours was exhausting | They were answering the question for three hours |
| 22 | attack | AmbV | 0.63% | 27.46% | The attack of the castle for three hours was grueling | They were attacking the castle for three hours |
| 23 | blame | AmbV | 0.43% | 15.09% | The blame of the criminal for three hours was unfounded | They were blaming the criminal for three hours |
| 24 | chase | AmbV | 0.30% | 27.48% | The chase of the criminal for three hours was successful | They were chasing the criminal for three hours |
| 25 | claim | AmbV | 3.03% | 30.02% | The claim of the seat for three hours was unsuccessful | They were claiming the seat for three hours |
| 26 | damage | AmbV | 0.74% | 43.53% | The damage of the car for three hours was expensive | They were damaging the car for three hours |
| 27 | hug | AmbV | 1.51% | 22.86% | The hug of the daughter for three hours was loving | They were hugging the daughter for three hours |
| 28 | kick | AmbV | 4.44% | 26.89% | The kick of the ball for three hours was strong | They were kicking the ball for three hours |
| 29 | kiss | AmbV | 3.91% | 23.42% | The kiss of the girl for three hours was sweet | They were kissing the girl for three hours |
| 30 | limit | AmbV | 10.02% | 58.15% | The limit of the texts for three hours was frustrating | They were limiting the texts for three hours |
| 31 | load | AmbV | 3.36% | 33.65% | the load of the car for three hours was exhausting | They were loading the car for three hours |
| 32 | offer | AmbV | 2.84% | 32.57% | The offer of the free drinks for three hours was unwelcome | They were offering the free drinks for three hours |
| 33 | praise | AmbV | 8.80% | 28.04% | The praise of the students for three hours was well-deserved | They were praising the students for three hours |
| 34 | promise | AmbV | 24.38% | 10.27% | The promise of free drinks for three hours was exciting | They were promising free drinks for three hours |
| 35 | protest | AmbV | 0.92% | 33.95% | The protest of the corrupt official for three hours was intense | They were protesting the corrupt official for three hours |
| 36 | request | AmbV | 15.26% | 46.42% | The request of the free drinks for three hours was annoying | They were requesting the free drinks for three hours |
| 37 | rescue | AmbV | 4.40% | 29.01% | The rescue of the daughter for three hours was successful | They were rescuing the daughter for three hours |
| 38 | reward | AmbV | 4.58% | 30.58% | The reward of the heroes for three hours was well-deserved | They were rewarding the heroes for three hours |
| 39 | support | AmbV | 23.78% | 45.30% | The support of the collapsing building for three hours was exhausting | They were supporting the building for three hours |
| 40 | visit | AmbV | 3.80% | 31.31% | The visit of the daughter for three hours was loving | They were visiting the daughter for three hours |

|  | **TABLE 2B: Experiment 1 Ambiguous Noun-base Stimuli (derived verbs)** | | | | | |
| --- | --- | --- | --- | --- | --- | --- |
|  |  |  | **Argument Structure COCA**  **frequency (mean)** | | **Alternate Argument Structure Acceptability Judgment** | |
| ***No*** | ***Item*** | ***Condition*** | ***# of "the __ of * NOUN" uses*** | ***# of "to ___ * NOUN" uses*** | ***We cleared the screen.*** | ***the screen cleared*** |
| 1 | brush | AmbN | 2.01% | 33.13% | We brushed our teeth. | the teeth brushed |
| 2 | buckle | AmbN | 14.66% | 2.41% | We buckled the seat. | the seat buckled |
| 3 | comb | AmbN | 2.11% | 49.59% | We combed her hair. | the hair combed |
| 4 | cover | AmbN | 12.10% | -1.17% | We covered the bed. | the bed covered |
| 5 | crack | AmbN | 10.31% | 2.11% | We cracked the glass. | the glass cracked |
| 6 | drill | AmbN | 0.08% | 20.54% | We drilled the metal. | the metal drilled |
| 7 | filter | AmbN | 7.84% | 21.98% | We filtered the coffee. | the coffee filtered |
| 8 | hook | AmbN | 0.64% | 14.63% | We hook the worm. | the wormed hooked |
| 9 | iron | AmbN | 0.17% | 20.08% | We ironed the clothes. | the clothes ironed |
| 10 | label | AmbN | 2.39% | 30.07% | We labeled the menu. | the menu labeled |
| 11 | lock | AmbN | 1.65% | 27.57% | We locked the house. | the house locked |
| 12 | mark | AmbN | 5.09% | 37.06% | We marked his name. | the name marked |
| 13 | match | AmbN | 1.08% | 41.24% | We matched the outfit. | the outfit matched |
| 14 | paint | AmbN | 0.27% | 30.44% | We painted the house. | the house painted |
| 15 | play | AmbN | 1.34% | 21.36% | We played the violin. | the violin played |
| 16 | recruit | AmbN | 0.00% | 29.51% | We recruited the students. | the students recruited |
| 17 | seal | AmbN | 8.08% | 43.21% | We sealed the bag. | the bag sealed |
| 18 | stamp | AmbN | 9.84% | 25.47% | We stamped the letter. | the letter stamped |
| 19 | switch | AmbN | 0.49% | 18.68% | We switched the light | the light switched |
| 20 | test | AmbN | 1.01% | 45.94% | We tested the products. | the products tested. |

Note: There are several noun-derived verbs that do, however, alternate in transitivity (e.g., crack) like those deadjectival verbs (e.g., clear), however, these verbs occur only in the Goal class/Figurative Goal of zero-derived verbs (see Tyler, L. J. 1999).

SUPPLEMENTARY TABLE 3:

| **TABLE 3: Experiment 2 (Eye Tracking) Stimuli** | | | |
| --- | --- | --- | --- |
| ***Item*** | ***Base Category*** | ***Derivation Status*** | ***Stimulus*** |
| 1 | Noun | Derived | Mary remembered to test for the learning disability. |
| 1 | Noun | Underived | Mary remembered the test for the learning disability. |
| 1 | Verb | Derived | Mary remembered the praise for the good work. |
| 1 | Verb | Underived | Mary remembered to praise for the good work. |
| 2 | Noun | Derived | John started to test after he found symptoms. |
| 2 | Noun | Underived | John started the test after he found symptoms. |
| 2 | Verb | Derived | John expected the visit after he found symptoms. |
| 2 | Verb | Underived | John expected to visit after he found symptoms. |
| 3 | Noun | Derived | Sarah planned to switch before the end of the summer. |
| 3 | Noun | Underived | Sarah planned the switch before the end of the summer. |
| 3 | Verb | Derived | Sarah planned the visit before the end of the summer. |
| 3 | Verb | Underived | Sarah planned to visit before the end of the summer. |
| 4 | Noun | Derived | Matthew started to drill while the carpenter sawed the wood. |
| 4 | Noun | Underived | Matthew started the drill while the carpenter sawed the wood. |
| 4 | Verb | Derived | Matthew started the chase when the whistle blew. |
| 4 | Verb | Underived | Matthew started to chase when the whistle blew. |
| 5 | Noun | Derived | Michelle remembered to paint behind the neighbor's shed. |
| 5 | Noun | Underived | Michelle remembered the paint behind the neighbor's shed. |
| 5 | Verb | Derived | Michelle remembered the visit with her neighbor. |
| 5 | Verb | Underived | Michelle remembered to visit with her neighbor. |
| 6 | Noun | Derived | Connor preferred to switch after the morning shift. |
| 6 | Noun | Underived | Connor preferred the switch after the morning shift. |
| 6 | Verb | Derived | Connor forgot the load near the door of the shop. |
| 6 | Verb | Underived | Connor forgot to load near the door of the shop. |
| 7 | Noun | Derived | David expected to drill behind his workshop. |
| 7 | Noun | Underived | David expected the drill behind his workshop. |
| 7 | Verb | Derived | David expected the answer before the student spoke. |
| 7 | Verb | Underived | David expected to answer before the student spoke. |
| 8 | Noun | Derived | Susan hated to paint because the color was ugly. |
| 8 | Noun | Underived | Susan hated the paint because the color was ugly. |
| 8 | Verb | Derived | Susan hated the answer during the lecture. |
| 8 | Verb | Underived | Susan hated to answer during the lecture. |
| 9 | Noun | Derived | Alfred liked to play since the actors were talented. |
| 9 | Noun | Underived | Alfred liked the play since the actors were talented. |
| 9 | Verb | Derived | Alfred remembered the answer when the teacher called. |
| 9 | Verb | Underived | Alfred remembered to answer when the teacher called. |
| 10 | Noun | Derived | Jane attempted to mark when the runners started. |
| 10 | Noun | Underived | Jane attempted the mark when the runners started. |
| 10 | Verb | Derived | Jane started the attack while the runners started. |
| 10 | Verb | Underived | Jane started to attack while the runners started. |
| 11 | Noun | Derived | Sam needed to paint since the house looked old. |
| 11 | Noun | Underived | Sam needed the paint since the house looked old. |
| 11 | Verb | Derived | Sam needed the visit with his old neighbor. |
| 11 | Verb | Underived | Sam needed to visit with his old neighbor. |
| 12 | Noun | Derived | Carolyn began to play while the customers clapped. |
| 12 | Noun | Underived | Carolyn began the play while the customers clapped. |
| 12 | Verb | Derived | Carolyn forgot the attack after the customers entered the shop. |
| 12 | Verb | Underived | Carolyn forgot to attack while the customers entered the shop. |
| 13 | Noun | Derived | Anna preferred to switch with the co-worker. |
| 13 | Noun | Underived | Anna preferred the switch with the co-worker. |
| 13 | Verb | Derived | Anna preferred the praise since other methods were ineffective. |
| 13 | Verb | Underived | Anna preferred to praise since other methods were ineffective. |
| 14 | Noun | Derived | Sarah planned to test for the learning disability. |
| 14 | Noun | Underived | Sarah planned the test for the learning disability. |
| 14 | Verb | Derived | Sarah planned the visit before the customer called. |
| 14 | Verb | Underived | Sarah planned to visit before the customer called. |
| 15 | Noun | Derived | Fred preferred to switch after the lecture. |
| 15 | Noun | Underived | Fred preferred the switch after the lecture. |
| 15 | Verb | Derived | Fred preferred the answer during the lecture. |
| 15 | Verb | Underived | Fred preferred to answer during the lecture. |
| 16 | Noun | Derived | Jenna attempted to mark when the runners started. |
| 16 | Noun | Underived | Jenna attempted the mark when the runners started. |
| 16 | Verb | Derived | Jenna preferred the praise since other methods were ineffective. |
| 16 | Verb | Underived | Jenna preferred to praise since other methods were ineffective. |
| 17 | Noun | Derived | James preferred to test after he found symptoms. |
| 17 | Noun | Underived | James preferred the test after he found symptoms. |
| 17 | Verb | Derived | James preferred the praise since other methods were ineffective. |
| 17 | Verb | Underived | James preferred to praise since other methods were ineffective. |
| 18 | Noun | Derived | Mark forgot to paint behind the doctor's office. |
| 18 | Noun | Underived | Mark forgot the paint behind the doctor's office. |
| 18 | Verb | Derived | Mark expected the visit after the doctor called. |
| 18 | Verb | Underived | Mark expected to visit after the doctor called. |
| 19 | Noun | Derived | Michael hated to paint because the color was dull. |
| 19 | Noun | Underived | Michael hated the paint because the color was dull. |
| 19 | Verb | Derived | Michael expected the answer because the student was dull. |
| 19 | Verb | Underived | Michael expected to answer because the student was dull. |
| 20 | Noun | Derived | Leah started to drill while the carpenter entered the shop. |
| 20 | Noun | Underived | Leah started the drill while the carpenter entered the shop. |
| 20 | Verb | Derived | Leah started the attack while the carpenter entered the shop. |
| 20 | Verb | Underived | Leah started to attack while the carpenter entered the shop. |
| 21 | Noun | Derived | Robert began to play when the whistle blew. |
| 21 | Noun | Underived | Robert began the play when the whistle blew. |
| 21 | Verb | Derived | Robert began the chase when the whistle blew. |
| 21 | Verb | Underived | Robert began to chase when the whistle blew. |
| 22 | Noun | Derived | Laura began to play while the audience clapped. |
| 22 | Noun | Underived | Laura began the play while the audience clapped. |
| 22 | Verb | Derived | Laura remembered the answer while the audience clapped. |
| 22 | Verb | Underived | Laura remembered to answer while the audience clapped. |
| 23 | Noun | Derived | Rachel needed to paint since the house looked old. |
| 23 | Noun | Underived | Rachel needed the paint since the house looked old. |
| 23 | Verb | Derived | Rachel needed the load near the old house. |
| 23 | Verb | Underived | Rachel needed to load near the old house. |
| 24 | Noun | Derived | Henry forgot to drill behind his workshop. |
| 24 | Noun | Underived | Henry forgot the drill behind his workshop. |
| 24 | Verb | Derived | Henry forgot the attack behind his workshop. |
| 24 | Verb | Underived | Henry forgot to attack behind his workshop. |

**Experiment 2 Stimuli Acceptability Judgements**

The stimuli created for Experiment 2 were rated by 80 native English speakers on Amazon’s Mechanical Turk, in order to ensure that the observed effect of derivation did not arise due to differences in acceptability across conditions. The conditions which exhibited wider variability in acceptability were the underived conditions; as a result, if there was a reading time slowdown incurred by sentence unacceptability, the two effects of sentence acceptability and derivation would apply to opposite sets of conditions, resulting in a smaller observed effect size for derivation. Thus, the significant effect of derivation observed in Experiment 2 is still reliable.


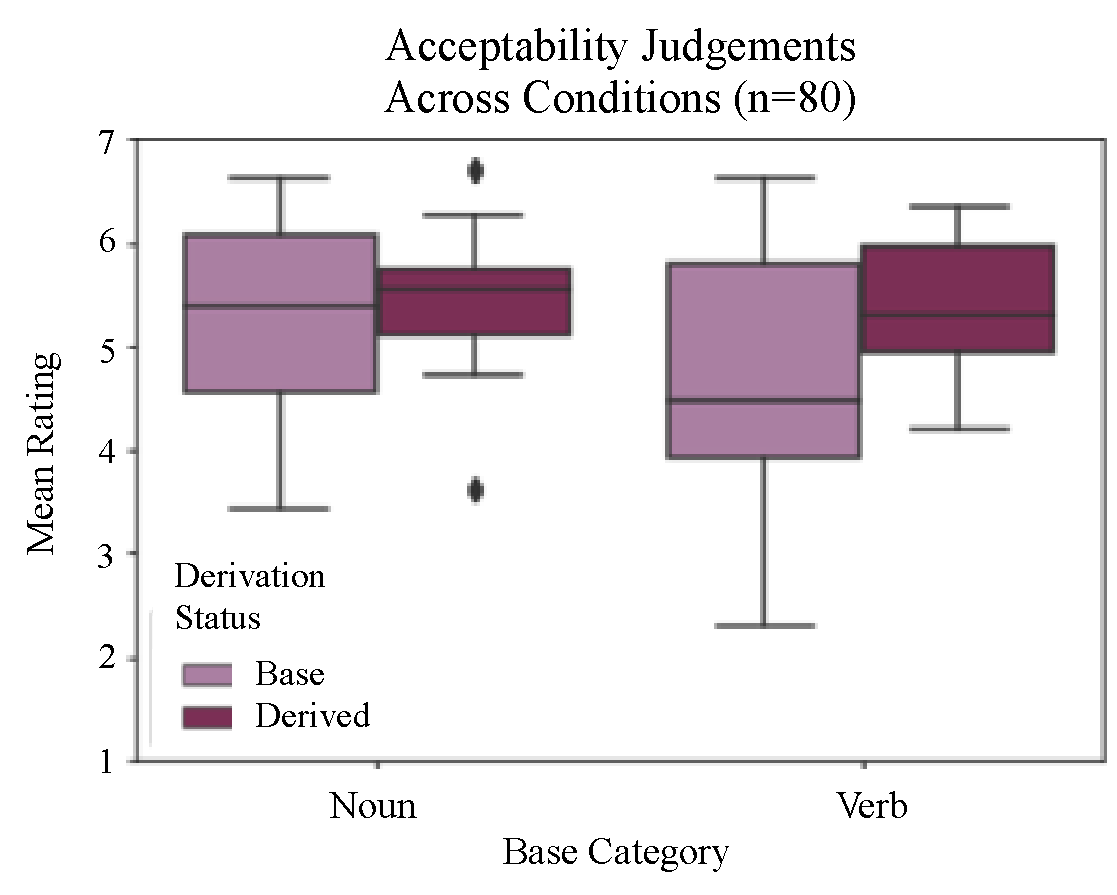


SUPPLEMENTARY FIGURE 1: Mean acceptability ratings (1: ‘least acceptable’, 7: ‘completely acceptable’) for the sentence stimuli used in Experiment 2.

To address any concerns of sentence acceptability having an effect on reading times at the critical region, another LMER model was fitted which included mean acceptability rating as a fixed effect. Importantly, this model did not make different conclusions from the earlier model (see Table 7) in regard to the effect of derivation, as significance was attained at the same (or higher) levels for the same factors in the same dependent measures. From this, we can conclude that derivation is the driving predictor for this effect and not sentence (un)acceptability.

SUPPLEMENTARY TABLE 4: Results of linear mixed effects regression model for Experiment 2 at the critical region, with a fixed effect of derivation nested within levels of base category, and a fixed effect of mean acceptability judgment rating, with random intercepts for subject and item.

| *Fixation Type* | *Derivation:BaseCategory* | *Estimate (Std. Err)* | *Pr (t)* |
| --- | --- | --- | --- |
| **First Fixation** | *Base:Noun* | 5.536 (0.089) | - |
|  | *Base:Verb* | 5.589 (0.083) | - |
|  | *Mean Rating* | -0.024 (0.015) | 0.129 |
|  | *Derived:Noun* | 0.086 (0.038) | 0.030* |
|  | *Derived:Verb* | 0.003 (0.039) | 0.519 |
| **First Pass** | *Base:Noun* | 5.544 (0.093) | - |
|  | *Base:Verb* | 5.607 (0.087) | - |
|  | *Mean Rating* | -0.024 (0.016) | 0.154 |
|  | *Derived:Noun* | 0.091 (0.039) | 0.021* |
|  | *Derived:Verb* | -0.019 (0.040) | 0.631 |
| **Regression Path** | *Base:Noun* | 5.930 (0.137) | - |
|  | *Base:Verb* | 6.004 (0.128) | - |
|  | *Mean Rating* | -0.049 (0.024) | 0.046* |
|  | *Derived:Noun* | 0.048 (0.058) | 0.441 |
|  | *Derived:Verb* | 0.171 (0.060) | 0.004** |
| **Total Time** | *Base:Noun* | 6.263 (0.139) | - |
|  | *Base:Verb* | 6.262 (0.129) | - |
|  | *Mean Rating* | -0.099 | 0.0001*** |
|  | *Derived:Noun* | 0.084 (0.058) | 0.145 |
|  | *Derived:Verb* | -0.017 (0.059) | 0.769 |
